# Characterisation of the Fibroblast Growth Factor Dependent Transcriptome in Early Development

**DOI:** 10.1371/journal.pone.0004951

**Published:** 2009-03-31

**Authors:** Peter A. Branney, Laura Faas, Sarah E. Steane, Mary Elizabeth Pownall, Harry V. Isaacs

**Affiliations:** Department of Biology, University of York, York, United Kingdom; INSERM, France

## Abstract

**Background:**

FGF signaling has multiple roles in regulating processes in animal development, including the specification and patterning of the mesoderm. In addition, FGF signaling supports self renewal of human embryonic stem cells and is required for differentiation of murine embryonic stem cells into a number of lineages.

**Methodology/Principal Findings:**

Given the importance of FGF signaling in regulating development and stem cell behaviour, we aimed to identify the transcriptional targets of FGF signalling during early development in the vertebrate model *Xenopus laevis*. We analysed the effects on gene expression in embryos in which FGF signaling was inhibited by dominant negative FGF receptors. 67 genes positively regulated by FGF signaling and 16 genes negatively regulated by FGF signaling were identified. FGF target genes are expressed in distinct waves during the late blastula to early gastrula phase. Many of these genes are expressed in the early mesoderm and dorsal ectoderm. A widespread requirement for FGF in regulating genes expressed in the Spemann organizer is revealed. The FGF targets MKP1 and DUSP5 are shown to be negative regulators of FGF signaling in early *Xenopus* tissues. *FoxD3* and *Lin28*, which are involved in regulating pluripotency in ES cells are shown to be down regulated when FGF signaling is blocked.

**Conclusions:**

We have undertaken a detailed analysis of FGF target genes which has generated a robust, well validated data set. We have found a widespread role for FGF signaling in regulating the expression of genes mediating the function of the Spemann organizer. In addition, we have found that the FGF targets MKP1 and DUSP5 are likely to contribute to the complex feedback loops involved in modulating responses to FGF signaling. We also find a link between FGF signaling and the expression of known regulators of pluripotency.

## Introduction

Fibroblast growth factors (FGFs) are small polypeptides that have multiple functions in early development and homeostasis of the adult organism. FGFs are present in all animal groups and are one of relatively few families of extracellular signaling molecules that are involved in regulating animal development. 22 FGFs have been identified in higher vertebrates [1].

FGF signaling has a key role in specifying the primary germ layers that give rise to all the tissues of the adult organism. Experiments initially carried out in amphibians, and later supported by studies in mammals, birds and fish, demonstrated that FGF signaling is required to regulate gene expression within the early vertebrate mesoderm, which is the germ layer giving rise to muscle, skeleton, connective tissue, blood and organs such as the kidney [2]–[6]. As well as regulating mesodermal gene expression, FGF signaling is involved in regulating the complex morphogenetic activity exhibited by mesoderm cells during vertebrate gastrulation [3], [7]. FGF signals produced by the mesoderm, acting on the adjacent ectoderm, are also required for induction and patterning of the vertebrate nervous system [8]–[11].

More recently it has been shown that FGF signaling plays a critical role in the commitment of mouse embryonic stem (ES) cells to mesodermal, as well as both neural and non-neural ectodermal lineages [12], [13]. FGF signaling is also important for maintaining self renewal in human ES and induced pluripotential stem (iPS) cells in culture [14]–[16].

Given the importance of FGF signaling in adult and embryonic life, the downstream transcriptional targets involved in mediating the activities of the FGFs are of great interest. In order to identify genes that respond to FGF signaling in early development we have compared gene expression in normal embryos with embryos in which FGF signaling has been inhibited. Our analysis identifies 67 genes which are significantly down regulated and 16 genes which are up regulated in response to FGF inhibition. A high proportion of the putative FGF target genes have predicted functions associated with cell signaling and transcriptional regulation.

We show that many of the targets are expressed in known regions of FGF activity during development. Our analysis reveals some interesting features of the FGF dependent transcriptome. We find that FGF signaling is required for the normal expression of multiple genes in the Spemann organizer, a structure orthologous to the node of higher vertebrates and which is required for the establishment of the main body axis.

Intrestingly, we find that inhibition of FGF signaling down regulates expression of *Lin28* and *FoxD3*, two genes which have been implicated in regulating the pluripotential state of embryonic stem (ES) cells [14], [17]–[19].

Cluster analysis, based upon temporal expression, identified a number of distinct waves of expression from FGF targets following the initial activation of FGF signaling in the amphibian embryo. Following the activation of endogenous FGF signalling in blastula stages, we show that two of the earliest expressed target genes are the *MAP kinase phosphatase 1* (*MKP1*) gene and a novel *Xenopus* gene related to human *Dual Specificity Phosphatase 5* (*DUSP5*). We show that both *DUSP5* and *MKP1* inhibit FGF dependent ERK/MAP kinase phosphorylation and mesoderm formation induced by FGF in *Xenopus* tissues. Our analysis indicates that *DUSP5* and *MKP1* are members of the FGF synexpression group and are components of the negative feedback network required to limit the extent of FGF signaling in the early embryo.

## Results

### Timing of FGF signaling in the Xenopus embryo

**Figure 1:**
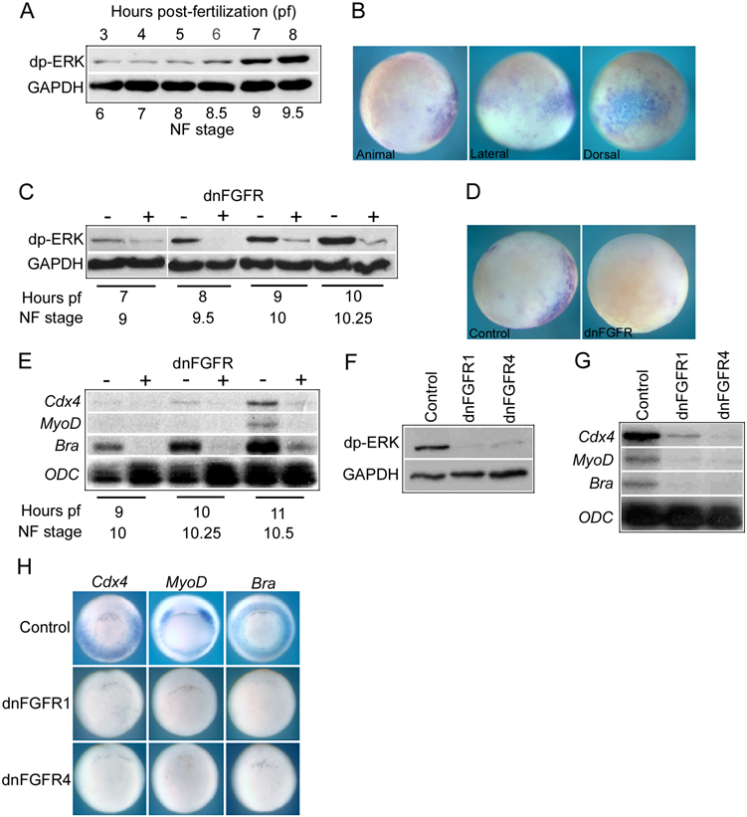
FGF signaling in early development. (A) is a Western blot showing levels of diphospo-ERK (dp-ERK) in whole embryos from cleavage stage 6 to late blastula stage 9.5 (3 to 8 hours pf at 23°C). GAPDH is a ubiquously expressed loading control. (B) shows whole mount immunohistochemistry for dp-ERK in blastula stage 9 embryos. In animal hemisphere view dorsal side is to the right. In lateral view the animal hemisphere is to the top and the dorsal side is to the right. In dorsal view the animal hemisphere is to the top. (C) is a Western blot comparing dp-ERK levels in control uninjected embryos and embryos injected with 4 ng dnFGFR4 mRNA from blastula stage 9 to early gastrula stage 10.25. (D) shows whole mount immunohistochemistry for dp-ERK at blastula stage 9 in a control uninjected embryo and an embryo injected with 4 ng dnFGFR4 embryo. Embryos are viewed from the animal hemisphere with dorsal side to the right. (E) is an RNAase protection analysis (RPA) showing the expression of *Cdx4*, *MyoD*, *brachyury* and ODC in control uninjected embryos and embryos injected with 4 ng dnFGFR4 mRNA from early gastrula stage 10 until stage 10.5. ODC is a ubiquioulsy expressed loading control. 10 µg of total RNA were used per hybridization. (F) is a Western blot showing dp-ERK levels in control uninjected embryos, embryos injected with 4 ng dnFGFR1 mRNA and embryos injected with 4 ng dnFGFR4 mRNA at early gastrula stage 10.5. The embryos are siblings to one set of the three biological replicates used for the microarray analysis. (G) is an RPA showing expression of *Cdx4*, *MyoD* and *brachyury* in control uninjected embryos, embryos injected with 4 ng dnFGFR1 mRNA and embryos injected with 4 ng dnFGFR4 mRNA at early gastrula stage 10.5. The embryos are siblings to one set of the three biological replicates used for the microarray analysis. (H) shows whole mount in situ hybridizations for *Cdx4*, *MyoD* and *brachyury* in control uninjected embryos , embryos injected with 4 ng dnFGFR1 mRNA and embryos injected with 4 ng dnFGFR4 mRNA at early gastrula stage 10.5. The embryos are siblings to one set of the three biological replicates used for the microarray analysis.

The aim of this study was to identify FGF targets that are induced shortly after the initial activation of zygotic FGF signaling in the embryo. It was therefore necessary to accurately determine when FGF signaling is activated in the embryo. We examined the temporal profile and spatial distribution of activated diphospho-ERK (dp-ERK), which is a key signal transduction effector of FGF signaling in the *Xenopus* embryo [20]–[22]. Figure 1A is a Western blot showing levels of dp-ERK in embryos from early cleavage to late blastula stages (NF stage 3 to 9.5). We detect constant low levels of activated ERK up to stage 8.5. The initial rise in the level of dp-ERK is detected at mid-blastula stage 8.5 and there is robust increase by stage 9 (7 hours post-fertilization (pf) at 23°C), corresponding to the onset of major zygotic transcription at the mid-blastula transition (MBT) [23].

Our data show that the initial activation of ERK is in a dorsal to ventral gradient within a broad belt of tissue around the equator of the embryo at late blastula stage 9 (Figure 1B). Initial dp-ERK activation is not limited to the presumptive mesoderm of the marginal zone but extends a considerable distance into the animal hemisphere on the dorsal side of the embryo. Figures 1C and D show that this early zygotic ERK activity is blocked by over expression of a dominant negative FGF receptor (dnFGFR). The dnFGFRs used in this study are carboxy-terminal truncations of the receptors lacking tyrosine kinase activity and block FGF signaling by associating with endogenous receptors to form non-functional dimers [24], [25]. We conclude that zygotic activation of the FGF signaling pathway commences at mid-blastula stage 8.5 (6 hours pf at 23°C).

### Timing of transcriptional responses to FGF signaling in the Xenopus embryo

The genes coding for the brachyury, MyoD and Cdx4 transcription factors have previously been identified as targets of the FGF signaling pathway. These genes are activated by FGF even in the presence of the translation inhibitor cycloheximide and are defined as immediate early responses to FGF signaling [26]–[28]. We have used expression of these genes to indicate when the initial transcriptional responses to FGF signaling occur in the *Xenopus* embryo. Figure 1E is an RNAase protection analysis (RPA) showing that *Brachyury* expression is detected by early gastrula stage 10 and *Cdx4* by stage 10.25. However, robust expression of all three immediate early FGF response genes is not detected until stage 10.5 (11 hours pf at 23°C). Furthermore, we show that the initial expression of all three genes is blocked by over expression of a dnFGFR. Based on the timing of ERK activation and transcriptional activation of known FGF target genes, early gastrula stage 10.5 (11 hours of culture at 23°C) was chosen as the stage for the analysis of FGF targets.

### Identifying transcriptional responses to FGF signaling

In order to identify transcriptional targets of FGF signaling gene expression in control embryos was compared to sibling embryos in which FGF signaling was inhibited by over expression of dominant negative versions of FGFR1 (dnFGFR1) or FGFR4 (dnFGFR4) [24], [25].

In order to undertake statistical analysis of the microarray data three biological replicates were carried out. Each replicate set consisted of control embryos and embryos injected with dnFGFR1 or dnFGFR4 collected at stage 10.5 (11 hours pf at 23°C). Before proceeding to microarray analysis each replicate set was checked for effective FGF inhibition. Sibling embryos from each replicate set were analysed for dp-ERK levels and expression of *Cdx4*, *Brachyury* and *MyoD* by both RPA and *in situ* hybridization. Figures 1F–1H are analyses of a representative biological replicate set showing that, with the experimental conditions used, overexpression of dnFGFR1 or dnFGFR4 results in potent inhibition of ERK activation (Figure 1F) and down regulation of transcription from known FGF target genes relative to sibling controls (Figures 1G and 1H).

### Changes in gene expression resulting from dnFGFR1 and dnFGFR4 overexpression

**Figure 2:**
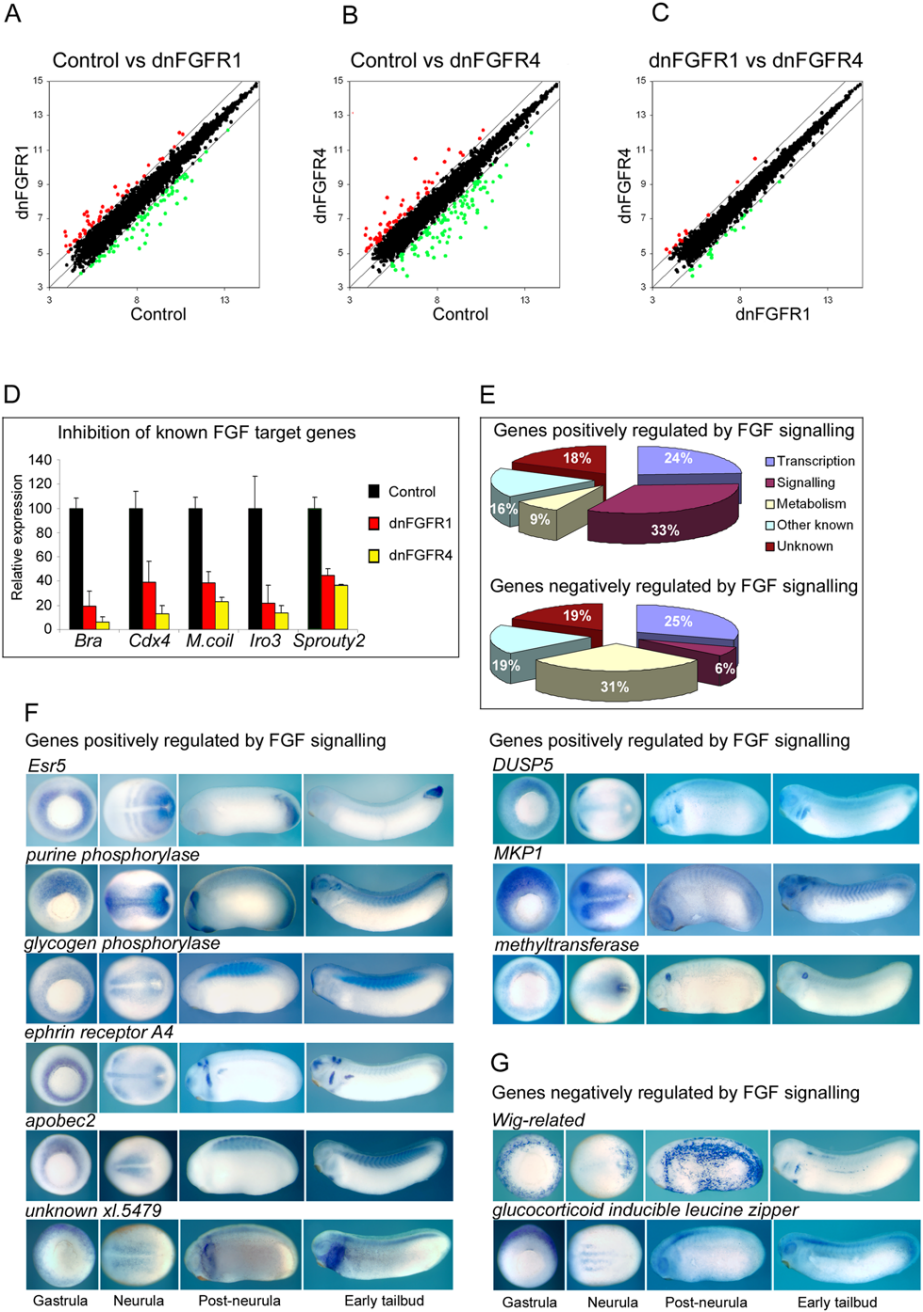
Identification of FGF target genes. (A, B and C) are scatterplots of log_2_ probeset expression values from the Affymetrix microanalysis undertaken on early gastrula stage 10.5 control embryos, dnFGFR1 injected embryos and dnFGFR4 injected embryos . Values for each point are the average of three biological replicates. The centre line represents a ratio of 1∶1 between the two groups indicating no difference in expression. The outlying lines represent two fold differences in expression. Points representing probe sets showing ≥2 reduction in expression are indicated in green. Points representing probesets showing ≥2 increase in expression are indicated in red. (D) is a chart showing the expression of *brachyury* (*bra*), *cdx4*, *marginal coil* (*M.coil*), *Iro3* and *sprouty2* in control embryos and embryos injected with 4 ng dnFGFR1 mRNA or 4 ng dnFGFR4 mRNA. Microarray derived expression values are based on the average of three biological replicates. Relative expression is calculated as a percentage of the expression in control embryos. Standard deviation bars are indicated. (E) shows pie charts of genes positively and negatively regulated by FGF signaling. Percentages of each group classified according to their putative cellular function are indicated. Details of the up regulated and down regulated genes are contained Tables 1, 2 and Tables S2 to S11. (F) shows the expression patterns of genes positively regulated by FGF signaling at determined by in situ hybridization at early gastrula stage 10.5, early neurula stage 14, post-neurula stage 22 and early tailbud stage 30. Gastrula embryos are vegetal views with dorsal to the top. Neurula embryos are dorsal views with anterior to the left. Post-neurula and tailbud embryos are lateral view with dorsal to the top and anterior to the left. (G) shows the expression patterns of genes negatively regulated by FGF signaling.

After these quality control checks, the RNA samples from each of the three biological replicates were analysed using Affymetrix GeneChip *Xenopus laevis* Genome Arrays, which allow the analysis of more than 14,400 transcipts expressed in early development. Figures 2A and 2B are scatterplots of log_2_ gene expression values in controls versus dnFGFR1 and dnFGFR4 injected embryos. Probe sets showing greater than 2-fold changes of expression in control versus experimental groups are indicated by red and green points. These data show that the expression of a considerable number of genes is altered in response to FGF inhibition by both dnFGFR1 and dnFGFR4.

**Table 1:** Genes positively regulated by FGF signaling.

Gene	Fold inhibition	GenBank accession	Affymetrix probe set
Brachyury	19.2	M77243	Xl.514.1.S1_at
Egr1	12.4	AF250345	Xl.637.1.A1_at
FoxD5A	10.8	AF162782	Xl.642.1.S1_at
SIP1	9.8	AB038353	Xl.958.1.S2_at
Cdx4	8.6	UO2034	Xl.10269.1.S1_at
Esr5	8.5	BJ057112	Xl.14524.1.S1_at
Purine phosphorylase	7.9	BM172525	Xl.16206.1.A1_at
Marginal coil	7.7	BJ044312	Xl.5454.1.S1_at
Paraxial protocadherin	7.3	AW782445	Xl.6173.1.A1_at
Glycogen phosphorylase	7.0	BJ056085	Xl.7815.1.A1_at
NADH dehydrogenase sub-unit	6.5	BJ051675	Xl.12993.1.A1_at
FoxD3A	6.0	AB014611	Xl.525.1.S1_at
G-coupled receptor P2Y5	5.7	BQ401062	Xl.19933.1.S1_at
Related to DC-STAMP domain receptor	5.6	BI447679	Xl.15270.1.A1_at
Meso05	5.1	BF615090	Xl.7720.1.A1_at
Uncharacterised protein C2orf32	5.1	CB756627	Xl.25136.1.A1_at
Frzb1	5.1	U78598	Xl.212.2.S1_a_at
XPO	5.0	BJ051206	Xl.5908.1.S1_s_at
Ephrin receptor A4	4.8	BJ080037	Xl.13.2.A1_at
XSpr2	4.5	BJ049843	Xl.2755.1.S1_a_at
Zic3a	4.5	AB005292	Xl.7969.1.S1_at
Xiro3	4.4	AF027175	Xl.4522.1.S1_at
Gravin-like	4.4	AF308810	Xl.3468.1.S1_at
Alkaline phosphatase	4.3	BC043760	Xl.1299.1.S1_at
Apobec2	4.2	AW766385	Xl.5876.1.A1_a
p75-like fullback receptor	4.2	AF131890	Xl.3540.1.S1_at
Wnt8	4.0	X57234	Xl.49.1.S1_at
Fructokinase-related protein	3.9	CB756273	Xl.15623.1.A1_at
Crescent	3.9	AF260729	Xl.619.1.S1_at
Pinhead	3.8	BJ056268	Xl.3529.1.A1_at
Wnt5b	3.6	AW148258	Xl.11619.1.S1_at
Unknown	3.6	BJ092401	Xl.5479.1.A1_at
Retrotransposon protein 1a11	3.6	L11263	Xl.3352.1.S1_at
FoxA4	3.4	S93559	Xl.1082.1.S1_at
Mitotic phosphoprotein 67	3.2	BJ077239	Xl.20772.1.A1_at
Cdx1	3.2	CB564190	Xl.23739.1.A1_at
Sprouty2	3.1	AF331825	Xl.11965.1.S1_at
DUSP5	3.0	BJ077463	Xl.15374.1.A1_at
Chordin	2.8	BF610870	Xl.3549.1.S1_at
MKP1	2.7	AJ320159	Xl.2803.1.S1_at
Unknown	2.7	BI312705	Xl.18179.1.S1_at
Xom	2.7	X98454	Xl.37.1.S1_at
Putative nucleolar GTP binding protein	2.7	BM179370	Xl.14776.1.A1_at
Lin28a homologue	2.7	BJ047699	Xl.3418.1.A1_at
Glut1 transporter	2.7	BJ049047	Xl.24121.1.A1_at
Unknown	2.6	BJ056692	Xl.15382.1.A1_at
Dkk1	2.6	AF030434	Xl.251.1.S1_at
Unknown	2.5	AW460550	Xl.11594.1.A1_at
RALDH2	2.5	BI449483	Xl.18999.1.A1_at
Prickle	2.5	AF387815	Xl.7556.1.S1_at
ADMP	2.4	BF231842	Xl.3809.1.A1_at
Unknown	2.3	BJ085271	Xl.1521.1.A1_at
Cytochrome B561	2.3	U16364	Xl.11917.1.S1_at
Goosecoid	2.3	BJ056432	Xl.801.1.S1_at
FoxC1	2.3	AF116844	Xl.180.1.S1_at
Noggin	2.2	M98807	Xl.834.1.S1_at
Sprouty1	2.2	BG022481	Xl.10087.1.A1_Fat
Oct1	2.2	BG022051	Xl.1265.1.S1_at
Rexp52	2.1	BG555868	Xl.3023.1.A1_at
Grb10 interacting protein2	2.1	BJ098841	Xl.14208.1.A1_at
Putative methyltransferase	2.1	BJ100128	Xl.20056.1.S1_a_at
Connexin 29	2.1	BJ076720	Xl.8924.1.A1_at
SMCT	2.1	BJ047968	Xl.6392.1.A1_at
Weakly similar to Rab1	2.1	BJ079872	Xl.3365.1.A1_at
Unknown	2.1	CA972457	Xl.19961.1.S1_at
Moderately similar to Brain protein 44	2.1	BJ088835	Xl.15887.1.S1_x_at
Ephrin receptor A2	2.0	BF025525	Xl.14496.1.A1_at

**Table 2:** Genes negatively regulated by FGF signaling.

Gene	Fold activation	GenBank accession	Affymetrix probe set
XIRG protein	13.5	AJ278067	Xl.4965.1.S1_at
PDGF A chain	5.6	M23238	Xl.841.3.S1_a_at
WIG-related	5.3	BJ044287	Xl.23988.1.S1_at
CP2-like transcription factor	4.3	BJ046394	Xl.16094.1.A1_at
Glucocorticoid inducible leucine zipper	4.1	BC043841	Xl.12378.1.S1_at
Unknown	3.8	AW147865	Xl.2077.1.A1_at
WIG	3.7	AF310008	Xl.736.1.S1_at
XANF1	3.1	X60099	Xl.131.1.S1_at
HES-related 1B	3.0	AB071434	Xl.12126.1.S1_at
Darmin	2.9	CD324819	Xl.6024.1.S1_at
ODC2	2.8	AF217544	Xl.8949.1.S1_at
Unknown	2.4	AW460608	Xl.11598.1.A1_at
Thioredoxin binding protein 2	2.3	BQ399899	Xl.24749.1.A1_at
Unknown	2.2	BM192746	Xl.25985.1.A1_at
Selenophosphate synthetase 1	2.1	BJ091471	Xl.6522.1.A1_at
Adenosine deaminase	2.1	BJ090126	Xl.24155.1.A1_at

In contrast to Figure 2A and 2B, in dnFGFR1 versus dnFGFR4 injected embryos we see that expression levels of relatively few probe sets are greater than two-fold different between the two groups (Figure 2C). Our analysis shows that only four genes, using the criteria of ≥2-fold change in expression and a significance level of p≤0.01, exhibit different expression levels in dnFGFR1 versus dnFGFR4 injected embryos (Table S1). This indicates that at this stage of development the genes affected by inhibition with the different dominant negative receptors are largely the same. The differences in gene expression profiles of dnFGFR1 and dnFGFR4 injected embryos are quantitative differences in the levels of expression from the same set of target genes. This conclusion is supported by Figure 2D, which shows that both dnFGFR1 and dnFGFR4 down regulate the expression of several known FGF targets. However, in all cases dnFGFR4 has a more potent effect on gene expression. The data in Figure 2A and 2B follow a similar trend, in which there are greater fold changes in gene expression following dnFGF4 injection than with dnFGFR1. We conclude that inhibition with dnFGFR1 and dnFGFR4 affects the same sets of genes but that on a per mass of injected mRNA basis, dnFGFR4 is more potent.

### Classification of putative FGF target genes

Lists of genes affected by FGF inhibition were compiled by comparing gene expression changes in dnFGFR4 injected embryos versus control embryos. The criteria of at least a 2-fold change in expression and a significance level of p≤0.01 were used to compile the gene lists. After the elimination of multiple probe sets representing the same gene, using the stated criteria, we find that 67 genes are significantly down-regulated by FGF inhibition, indicating that in normal development these genes are positively regulated by FGF signaling. Table 1 shows these genes in order of mean fold inhibition in dnFGFR injected embryos relative to controls. The T-box gene *brachyury* shows the highest fold inhibition (>19-fold). We find that only 16 genes are significantly up-regulated by FGF inhibition, indicating that in normal development these genes are negatively regulated by FGF signaling (Table 2).

Where possible we have classified FGF target genes based on cellular function. We find that a large proportion of genes positively regulated by FGF signaling are either involved in transcriptional regulation (24%) or cell signaling (18%), with a relatively small number of genes (9%) involved in various aspects of metabolism. The corresponding figures for genes negatively regulated by FGF signaling are 29% involved in transcriptional regulation, 6% in cell signaling and 29% in metabolism. These data are represented as pie charts in Figure 2E and the detailed breakdown of FGF target classification, together with relevant references are presented in Tables S2, S3, S4, S5, S6, S7, S8, S9, S10 and S11. Tables S12 and S13 show the Gene Ontology (GO) term classifications for FGF targets derived from the available Affymetrix annotation files.

### Expression of FGF target genes

Consistent with the pattern of FGF activity in the late blastula and early gastrula stage embryo (Figure 1) many of the genes identified as being down regulated following FGF inhibition are expressed in the mesoderm or dorsal ectoderm at the start of gastrulation. Expression data for previously characterised genes, along with new data from this study are summarized in Table S14.

We have undertaken a more detailed expression analysis of a number of these positively regulated FGF targets at early gastrula, early neurula, post-neurula and early tailbud stages. (Figure 2F) We find that all of these genes are expressed in the mesoderm around the mesoderm. In post-gastrula stages the expression patterns of these genes diversify; however, some common patterns are detected. For example, the posterior mesoderm, the paraxial mesoderm and the tailbud are common sites of expression. Other sites of expression include the anterior CNS and branchial arches. In the case of a putative methyltransferase the post-gastrula expression is remarkably restricted, being limited to a very tight domain around the closed blastopore and later in the developing otic vesicle.

The expression patterns were also determined for two of the genes that are up regulated in response to FGF inhibition, which we predict will be negatively regulated by FGF signaling in normal development (Figure 2G).

*Wig-related* codes for a protein similar to Xwig1, which is a putative endoplasmic reticulum resident protein [29]. GILZ (glucocorticoid inducible leucine zipper), is a member of the Tsc-22 family of transcription factors related to *Drosophila* bunched [30]. In contrast with genes positively regulated by FGF, the early expression of these genes is excluded from the circum-blastoporal region. The *Wig-related* gastrula and post-gastrula expression pattern is highly dynamic, before resolving to a stable pattern of expression in the CNS, branchial arches and lateral mesoderm. After gastrulation GILZ is expressed in the neurogenic region of the open neural plate and subsequently in the neural tube.

### Validation of FGF targets

**Figure 3:**
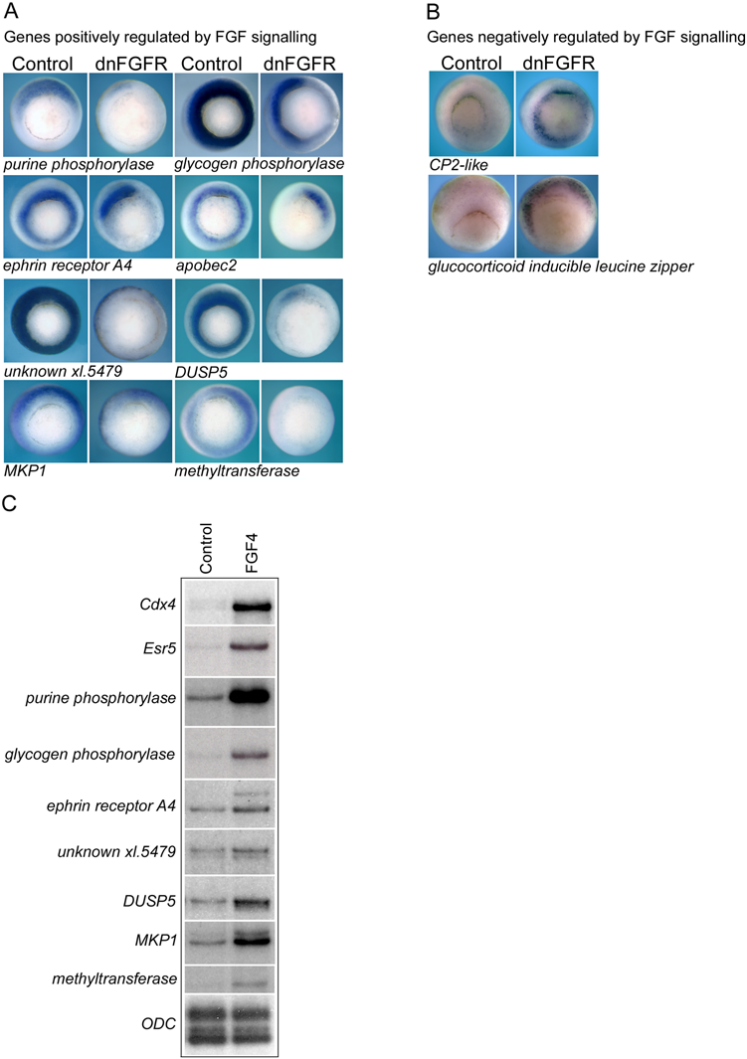
Validation of FGF target genes. (A) shows whole mount in situ hybridizations for genes positively regulated by FGF signaling at gastrula stage 10.5 in control embryos and embryos injected with 2 ng of dnFGFR1 mRNA. (B) shows the expression gene negatively regulated by FGF signaling in control embryos and embryos injected with 2 ng dnFGFR1 mRNA. All embryos are vegetal view with dorsal to the top. Non-uniform down regulation around the circumference of the blastopore is apparent in some embryos and is likely due to variability in the diffusion of injected dnFGFR mRNA. (C) is an RPA showing the expression at gastrula stage 10.5 of a number of genes in control animal cap explants and explants treated with FGF4 protein. 5 µg total RNA was used per hybridization. ODC is a loading control.

It is generally accepted that gene lists identified by microarray analysis should be validated by independent methodology. For a number of the putative target genes independent validation of their response to FGF signaling was undertaken by in situ hybridization. Figure 3A shows the effects of FGF inhibition on the spatial expression of genes identified as being down regulated in the microarray-based screen. Injection of dnFGFR leads to dramatic inhibition of the circum-blastoporal expression of these genes in gastrula stage embryos.

Conversely, the size of the expression domains of two putative targets negatively regulated by FGF signaling are dramatically increased in embryos injected with dnFGFR mRNA (Figure 3B). In the case of CP2-like and GILZ, inhibition of FGF signaling leads to elevated expression in the circum-blastoral region indicating that in normal development FGF signaling is required to exclude their expression from this region of the embryo.

Further validation of the FGF target genes was undertaken by showing that FGF signaling is not only necessary but is also sufficient for their expression. Figure 3C shows an RNAase protection assay (RPA) on control animal hemisphere tissue explants (animal caps) and animal caps treated with recombinant FGF4 protein. With all genes tested, FGF treatment leads to marked increase in transcription as compared to control explants. Taken together, our analyses indicate that the microarray based screen has provided a well supported list of candidate FGF target genes.

### FGF signaling and dorsal gene expression

**Figure 4:**
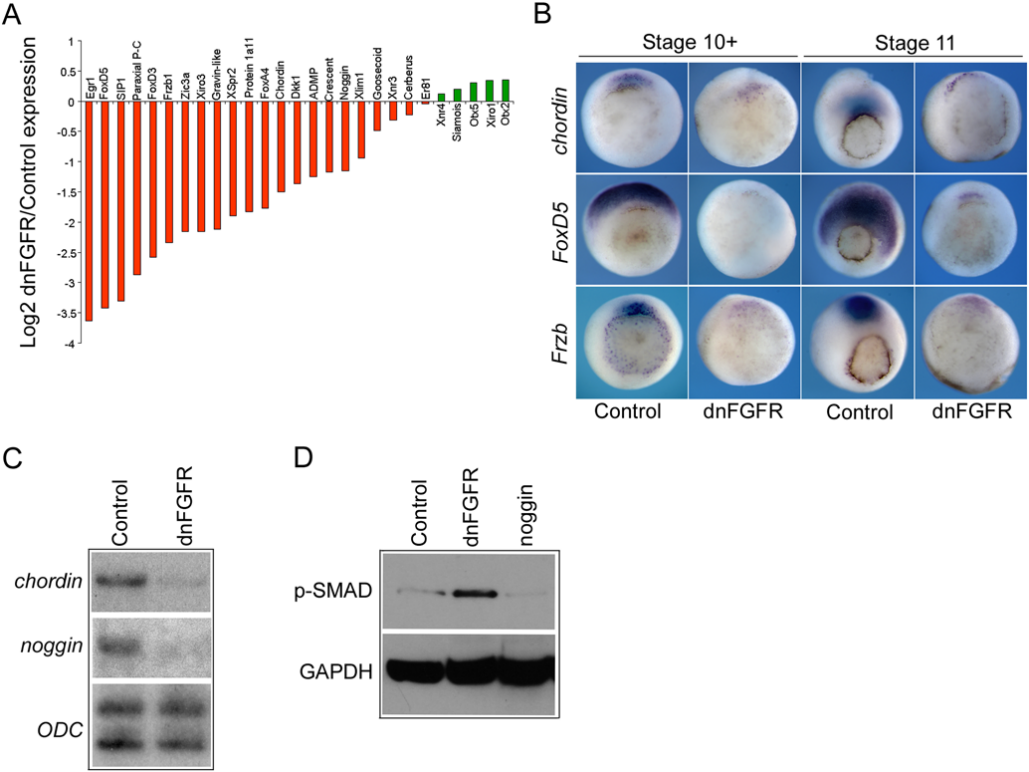
FGF signaling and regulation of dorsal gene expression. (A) is a bar chart showing the log_2_ of the ratio of expression dnFGFR4 injected embryos versus control embryos for a number of dorsally expressed genes at gastrula stage 10.5. Microarray derived expression values are based on the average of three biological replicates. Bars in red below the centre line represents genes down regulated in response to FGF inhibition. Bars in green represent genes up regulated in response to FGF inhibition. (B) shows whole mount in situ hybridizations for *chordin*, *FoxD5* and *Frzb* in control embryos and embryos injected with 4 ng dnFGF4 mRNA at very early gastrula stage 10+ and mid-gastrula stage 11. All embryos are vegetal views with dorsal to the top. (C) is an RPA showing the expression of *chordin* and *noggin* in control embryos and embryos injected with 4 ng dnFGFR1 mRNA at gastrula stage 10.5. (D) is a Western blot showing levels of phospho-SMAD1/5/8 (p-SMAD) at gastrula stage 10.5 in control embryos, embryos injected with 4 ng dnFGFR1 mRNA or 1 ng mRNA coding for secreted the BMP inhibitor noggin. GAPDH is a loading control.

The identified FGF target genes included several genes, including *chordin* and *noggin*, which are required for the function of the Spemann organizer in the dorsal mesoderm [31], [32]. This region of the embryo plays a key role in regulating the formation of the main body axes. We investigated if there is a general role for FGF in regulation of dorsal gene expression. The data in Data in Figure 4A and Table 3 show that many dorsally biased genes, including *Egr1* and *FoxD5* are highly down regulated when FGF signaling is inhibited (>12-fold and >10-fold respectively). Many of the dorsally expressed FGF target genes have been shown to be directly involved in mediating organizer function, including *Frzb1*, *chordin*, *noggin*, *FoxD3*, *FoxD5* and *goosecoid*. Other dorsally expressed genes, such as *Xnr3* and *cerberus*, are not significantly down regulated, and others such as *Otx2* show small increases in expression (not significant at the p = 0.01 level).

**Table 3:** Effects of FGF inhibition on organizer gene expression.

Gene	Ratio of expression in dnFGFR and control groups
Egr1	12.4
FoxD5A	10.8
SIP	9.8
Paraxial P-C	7.3
FoxD3A	6
Frzb1	5.1
Zic3A	4.5
Xiro3	4.5
Gravin-like	4.4
Crescent	3.9
Xspr2	3.7
Protein 1a11	3.6
FoxA4	3.4
Chordin	2.9
Dkk1	2.6
ADMP	2.4
Goosecoid	2.3
Noggin	2.2
Xlim1	1.9
Xnr3	1.2
Cerberus	1.2
Er81	1.0
Xnr4	0.9
Siamois	0.9
Otx5	0.8
Xiro1	0.8
Otx2	0.8

The *in situ* hybridizations in Figure 4B show that FGF inhibition dramatically reduces the spatial extend of *chordin*, *FoxD5* and *Frzb* expression through early gastrula stages. We also show by RPA that *chordin* and *noggin* are strongly down regulated in early gastrula stage embryos following FGF inhibition (Figure 4C), indicating a role for FGF in regulating the expression of secreted BMP inhibitors.

Stimulation of BMP signaling leads to the phosphorylation and activation of SMAD1. Figure 4D is a Western blot for phospho-SMAD1/5/8 (p-SMAD). In keeping with a role for FGF signaling in regulating dorsally expressed secreted BMP inhibitors, such chordin and noggin, levels of p-SMAD1/5/8 are elevated in response to FGF inhibition. Our data indicate a widespread but not ubiquitous requirement for FGF signaling in the regulation of organizer gene expression during gastrula stages.

### Expression profiling and cluster analysis of FGF target genes

In order to generate temporal expression profiles for individual FGF target genes from pre-MBT stages until early neurula stages we carried out Affymetrix microarray analysis on normally developing sibling embryos at hourly time points from 5 hours pf (stage 8) to 16 hours pf (stage 14) at 23°C.

**Figure 5:**
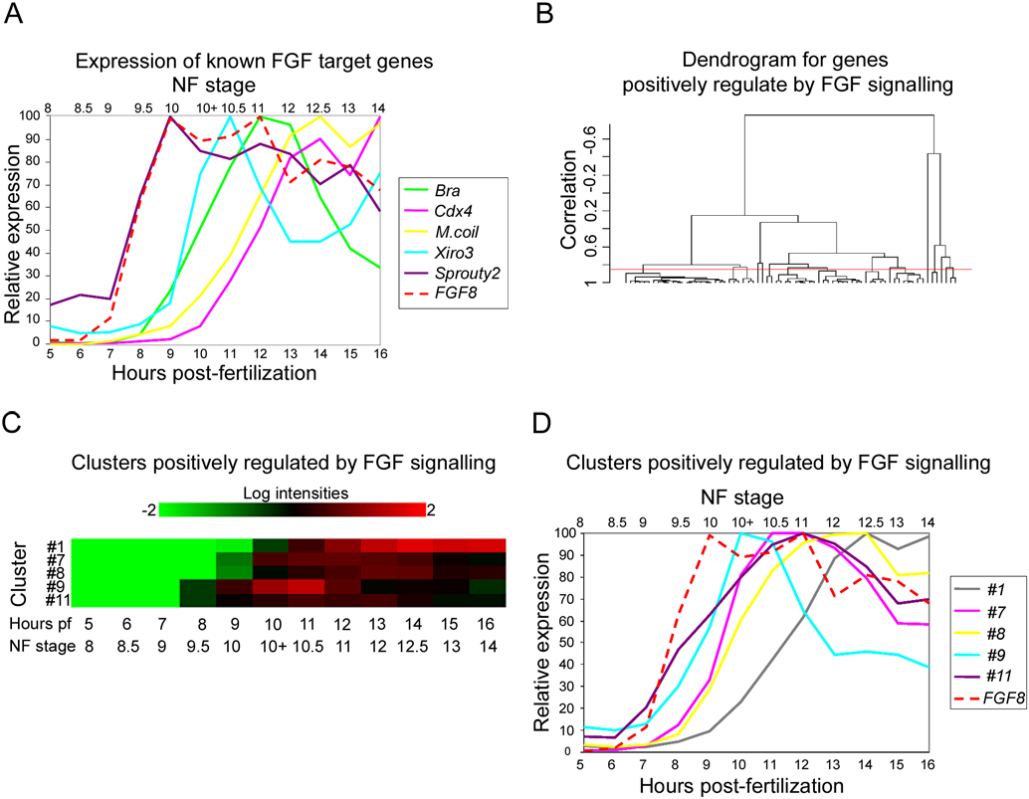
Cluster analysis of genes down regulated in response to FGF inhibition. (A) shows the temporal expression profiles of *FGF8* (dashed line) and several known FGF target genes from blastula stage 8 until early neurula stage 14 (5 to 16 hours pf at 23°C). Profiles are derived from normalised microarray expression levels. Relative expression values are represented as percentages of the maximum expression value for each gene. (B) is a cluster dendrogram generated within BRB-ArrayTools for genes that are significantly down regulated in response to FGF inhibition i.e. positively regulated by FGF signaling. The red line indicates the level at which the dendrogram was cut, corresponding to a correlation coefficient of 0.85. (C) is a heat map of temporal expression for gene clusters positively regulated by FGF signalling. Only clusters containing ≥5 members are presented. Values at each time point from blastula stage 8 to early neurula stage 14 are derived from the mean of the expression levels for all the genes in each cluster. (D) shows the temporal expression profiles of *FGF8* and gene clusters positively regulated by FGF signalling based upon the mean of the expression levels for all the genes in each cluster.

Figure 5A shows the relative expression profiles of *FGF8* and 5 known FGF target genes. The initial rise in *FGF8* expression is first detected at stage 9 (7 hours pf), indicating that *FGF8* expression is activated very rapidly post-MBT. As mentioned earlier, this increase in *FGF8* expression corresponds closely with the detected rapid elevation of dp-ERK levels in the embryo between stage 8.5 and stage 9 (Figure 1A). We note that although normal expression of these genes requires FGF signaling, the timing of gene activation relative to the initiation of FGF signaling in the embryo can be quite different. For example, *sprouty2* is expressed at low levels maternally and the initial rise in levels of zygotic expression is detected at stage 9.5 which is 1 hour at 23°C after the activation of *FGF8* expression. Subsequently, *sprouty2* expression continues to closely follow that of *FGF8* during late blastula stages (stage 9 to 10). The initiation of *brachyury* and *Iro3* expression is somewhat delayed relative to initiation of FGF signaling, with a slight rise in expression by stage 9.5 and a more significant increase in expression by stage 10. Expression from *Cdx4* and *marginal coil* are even more delayed and their expression levels only begin to rise steeply from stage 10 onwards.

The differing dynamics of expression from known target genes indicate that there are different classes of FGF response genes. We investigated this further by undertaking a cluster analysis of FGF dependent genes based upon their temporal expression profiles from early blastula to early neurula stages. For the generation of clusters of genes positively regulated by FGF signaling a correlation value of p≥0.85 was used. The cluster analysis of genes negatively regulated by FGF is not presented because this group contains considerably fewer genes leading to multiple clusters containing single genes.

The genes in each of the clusters used for further analysis are shown in Table 4. The dendrogram generated during cluster analysis is shown in Figure 5B. Figure 5C shows a heat map of the relative expression of each of the generated clusters from stage 8 to stage 14. The expression profiles of the clusters positively regulated by FGF signaling, together with that of *FGF8*, are shown in Figure 5D. In keeping with our findings for individual known FGF targets, the initiation of expression from each of the clusters relative to the activation of FGF signaling varies considerably. For example, activation of expression from genes in clusters #11 and #9 rapidly follows the activation of *FGF8* expression. However, at 23°C activation of expression from genes in cluster #1 is delayed 2–3 hours relative to that of *FGF8*. Activation of expression from genes in clusters #7 and #8 occurs at an intermediate time point with a 1–2 hour lag relative FGF8 and the activation of FGF signaling.

**Table 4:** Gene clusters positively regulated by FGF signaling.

Cluster	Gene
#1	Alkaline phosphatase
	Cdx4
	Cytochrome B561
	FoxC1
	Glycogen phosphorylase
	Gravin-like
	Lin28a homologue
	Marginal coil
	mitotic phosphoprotein 67
	p75-lke fullback receptor
	Purine phosphorylase
	Putative methyltransferase
	Unknown
	SIP1
	SMCT
	Uncharacterised protein C2orf32- xl.25136
	Unknown-xl.15382
#7	Dkk1
	FoxA4
	FoxD5A
	Frzb1
	NADH dehydrogenase sub-unit
	Pinhead
	Unknown-xl.3023
	XSpr2
	Zic3A
#8	Brachyury
	Chordin
	Ephrin receptor A4
	FoxD3A
	Glut1 transporter
	Paraxial protocadherin
	Retrotransposon protein 1a11
	Unkown-Xl.18179
	unkown-xl.5479
#9	Egr1
	FoxA4
	Goosecoid
	Prickle
	Related to DC-STAMP domain receptor
#11	ADMP
	DUSP5
	Esr5
	Noggin
	Sprouty2
	Wnt8
	Xom

### Identification of a novel negative regulator of FGF signaling

The earliest activation of zygotic transcription from putative FGF target genes occurs between blastula stage 8.5 to 9. During this period a number of genes undergo >10-fold increase in expression. Amongst these rapid responders *Dual specificity phosphatase 5* (*DUSP5*) has the highest fold increase during this period (>35). *Xenopus laevis DUSP5* codes for a putative MAP kinase phosphatase with 61% peptide sequence identity to human DUSP5 (Figure S1).

Expression of another MAP kinase phosphatase, *MKP1/XCL100* [33], is also significantly down regulated in response to FGF inhibition (Table 1 and Figure 3). Interestingly, another *Xenopus* MAP kinase phosphatase, MKP3, has been shown to inhibit FGF dependent mesoderm induction [21] and is implicated as a component of a negative feedback loop regulating FGF activity in the embryo [34]. Given the critical role that ERK/MAP kinase activity has in mediating responses to FGF signaling in the early *Xenopus* embryo we investigated the potential role of DUSP5 and MKP1 as negative regulators of FGF mediated MAP kinase signaling in early development.

**Figure 6:**
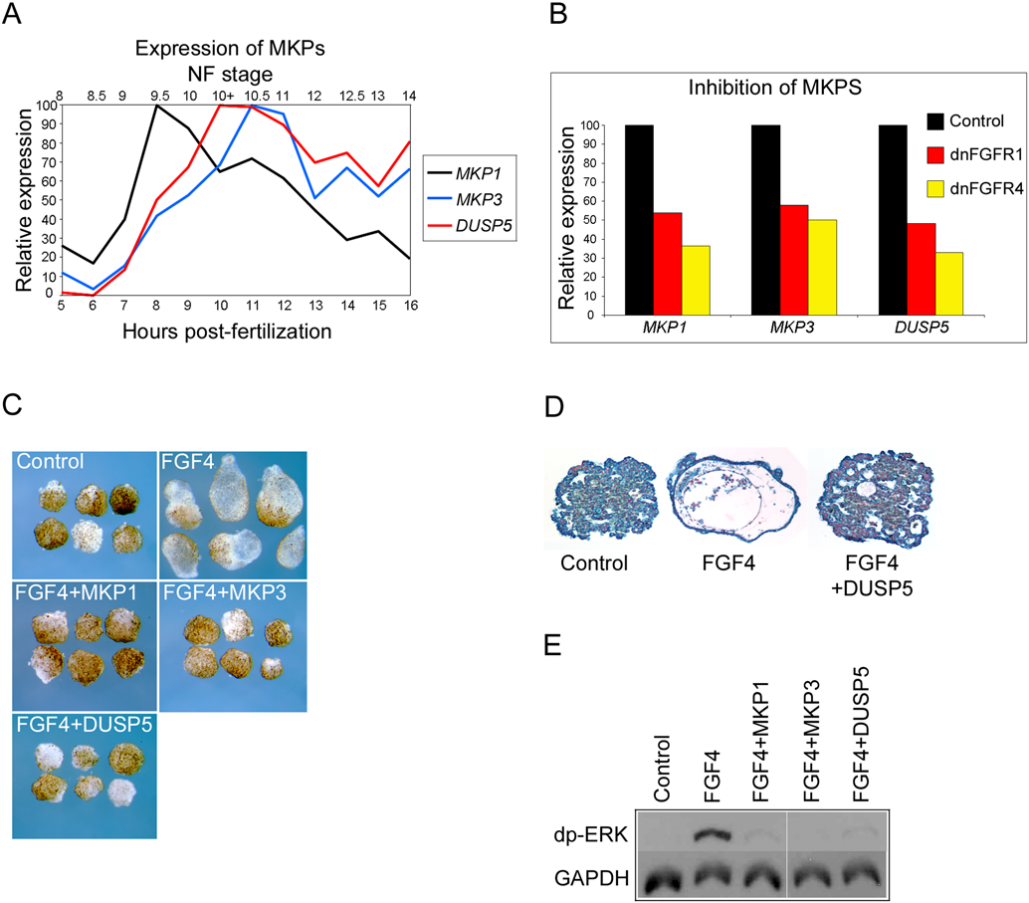
MKPs and FGF signalling. (A) shows the temporal expression profiles of *MKP1*, *MKP3* and *DUSP5* from blastula stage 8 until early neurula stage 14 (5 to 16 hours pf at 23°C). Profiles are derived from normalised microarray expression levels. Relative expression values are represented as percentages of the maximum expression value for each gene. (B) is a chart showing the expression of *MKP1*. *MKP3* and *DUSP5* in control embryos and embryos injected with 4 ng dnFGFR1 mRNA or 4 ng dnFGFR4 mRNA. Microarray derived expression values are based on the average of three biological replicates. Relative expression is calculated a percentage of the expression in control embryos. Experiments in (C, D and E) were carried out on animal cap explants removed from blastula stage 8 embryos. In all cases control explants are from uninjected embryos, FGF4 treatment was with 10 units of recombinant protein and mRNA injections were with 10 ng *MKP1*, *MKP3* or *DUSP5*. (C) shows the morphology of animal cap explants at tailbud stage 41. (D) shows 10 µm histological sections of animal cap explants at stage 41. (E) is a Western blot showing levels of dp-ERK and the loading control GAPDH in animal cap explants at stage 10.5.

Figure 6A is a chart showing the temporal expression profiles of *MKP1*, *MKP3* and *DUSP5* as determined by microarray analysis. Expression of all three MKPs rises rapidly during late blastula stages, reaching maxima in early gastrula stages. Our data show that both *MKP1* and *DUSP5* are expressed in the circum-blastoporal region of the embryo during gastrula stages (Figures 2F). *MKP3* is also expressed in this region in the gastrula [34]. In post-gastrula stages *MKP1* is expressed in much of the open neural plate and subsequently in the anterior CNS and somites (Figure 2F). In contrast, at neurula stages *DUSP5* is expressed in the posterior mesoderm around the closed blastopore and in a restricted domain in the anterior open neural plate. Later in development it is expressed in distinct domains in the anterior CNS, the tailbud and branchial arches.

Figure 6B shows the degree to which expression of the three MKPs in the early gastrula is down regulated in response to FGF inhibition with dnFGFR1 and dnFGFR4. Consistent with this, expression of *MKP1* and *DUSP5* in the circum-blastoporal region during gastrula stages is dependent on FGF signaling (Figure 3A). Similar FGF dependence has been reported for *MKP3* [34]. We also show that FGF signaling is sufficient for *MKP1* and *DUSP5* expression; treatment of animal cap explants with FGF proteins leads to marked up regulation of both genes (Figure 3C).

Treatment of animal cap explants from blastula stage embryos with FGF protein results elongation of the explant and development of vesicles containing a range of mesodermal tissues. Figure 6C shows that overexpression of *MKP1*, *MKP3* or *DUSP5* inhibits the formation of vesicles, indicating that like MKP3, MKP1 and DUSP5 are able to block FGF mediated formation of mesodermal tissues. We have confirmed this by examining histology of the differentiated tissues in FGF and DUSP5 treated explants (Figure 6D). Sections of untreated, control explants show the presence of a mass of atypical epidermis, whereas, FGF treated explants contain copious mesodermal tissue types, including a layer of smooth muscle (mesothelium) and loosely packed mesenchyme. Histology reveals the absence of mesodermal differentiation in response to FGF treatment when explants are over expressing DUSP5. Figure 6E shows that treatment of animal cap explants with FGF4 protein leads to phosphorylation and activation of ERK/MAP kinase and that over expression of either MKP1, MKP3 or DUSP5 in animal hemisphere explants dramatically inhibits FGF induced ERK phosphorylation.

Our data indicate that normal expression of *MKP1* and *DUSP5* in the early embryo requires a functional FGF pathway and that MKP3, MPK1 and DUSP5 have similar abilities to negatively regulate FGF signaling.

## Discussion

### FGF signaling in the early embryo

There is a wealth of evidence indicating that FGF signalling is involved in regulating multiple developmental processes before and during amphibian gastrulation [1], [35]. FGF dependent regulatory pathways have been shown to operate at different levels within the cell. For example, FGF signal transduction involving PKC and Ca++ modulates the planar cell polarity pathway necessary for the morphogenetic movements of gastrulation [36]. Another key function of FGF signalling during gastrula stages is as a regulator of gene transcription and it is this latter function which is the focus of the present study.

### Identification of transcriptional targets of FGF signaling

Our study was designed to identify early transcriptional responses to FGF signalling. The reagents that we used to inhibit FGF signaling were dominant negative mutants of FGF receptor 1 (dnFGFR1) and FGF receptor 4a (dnFGFR4a) [24], [25]. Over expression of either dnFGFR1 or dnFGFR4a leads to very similar effects on gene expression at the start of gastrulation.

Using the strict criteria outlined, we have identified 67 genes which are down regulated and 16 genes which are up regulated in response to FGF signaling. The target validation undertaken indicates that these FGF targets lists are well supported and should provide the basis for further studies into FGF dependent transcriptional regulation.

### Different waves of FGF dependent gene regulation

As part of this study we carried out a time course analysis of gene expression from mid-blastula to early neurula stages. Our cluster analysis based on these expression profiles reveals that activation of expression from FGF dependent genes occurs in a number of waves following the mid-blastula transition (MBT). Some genes are activated very rapidly following the MBT and closely follow the expression profile of *FGF8*. Expression of other genes, including *Brachyury* and *Cdx4*, which are known immediate early response genes, activated by FGF signaling in the absence of protein synthesis, occurs in later waves [26], [37]. At present it is unclear why some immediate early responses are more rapid than others. However, we speculate that the presence of identifiable clusters of FGF response genes indicates that similar upstream mechanisms are involved in regulating the expression of genes within the same cluster. The identification of such clusters of co-expressed transcriptional targets of FGF signaling will allow the analysis of these genes for shared regulatory elements required to drive their common modes of expression.

It is important to note that our analysis does not rule out the involvement of other signaling pathways in the regulation of the identified FGF target genes. Indeed this is to be expected, given that FGF signaling has been shown to interact with a number of pathways regulating gene expression in early development, including the activin/nodal and Wnt signaling pathways [38]–[41].

### Patterns of FGF target gene expression

The initial zygotic expression of several FGF ligand genes, including *FGF3*, *FGF4*, *FGF8* [42]–[44] and *FGF20* (Figure S2) is restricted to the early mesoderm during late blastula stages. In keeping with this we find that many target genes positively regulated by FGF signaling are also expressed in the mesoderm. Our analysis of FGF dependent gene expression in later development shows that there is diversity in their later expression. However, we note that the posterior mesoderm, the paraxial mesoderm, the branchial arches and tailbud are common sites of expression for the FGF target genes. These are all known sites of FGF activity and ligand expression including *FGF3*, *FGF4* and *FGF8* [22], [42]–[44]. We show that *FGF7* and *FGF10* are also expressed in the posterior of the embryo and in the branchial arch region (Figure S2). An interesting example of the correspondence of target gene expression with sites of FGF signaling in later development is seen with a putative *methyltransferase* gene, which in tailbud stages is expressed exclusively in the developing otic vesicle in close proximity to *FGF10* expression (Figure S2).

### FGF regulation of organizer gene expression

Early studies of FGF function in early amphibian development focused on their potential role as regulators of gene expression in the ventro-lateral mesoderm [35], [45], [46]. However, more recent studies have provided evidence that FGF signaling is also required for the expression of genes within the dorsal organiser region of the amphibian embryo [47]–[49]. The large scale analysis of gene expression provided by our microarray experiments show that FGF signaling is required for the normal expression of a large number of organizer genes, including *goosecoid*, *chordin*, *noggin*, *dkk1* and *Frzb*, and a number of genes, such as *Sip1* and *FoxD5*, which are expressed in the dorsal neuroectoderm (See Table S14). Such a role is very much in keeping with the observed activity of MAP kinase signalling in the dorsal marginal zone and dorsal neuroectoderm, and supports the view that FGF signaling is required during late blastula and early gastrula stages for the establishment of both the Spemann organizer and the presumptive neuroectoderm.

There are also indications that FGF signaling is required to negatively regulate and therefore restrict gene expression in the dorsal region of the embryo. For example, the transcription factors *Hes1b* is up regulated in response to FGF inhibition. We note that *Hes1b* is expressed in the dorsal region of the gastrula but at some distance from the highest levels of FGF activity in the blastopore region [50]. It is also seems likely that negative regulation by FGF restricts *XANF1* expression to the deeper layers of the organizer region in the early gastrula [51].

### Gene function downstream of FGF signaling

A detailed description of the putative function for each of the identified target genes is beyond the scope of this discussion and we refer the reader to the extensive annotation and literature resources provided in Tables S2, S3, S4, S5, S6, S7, S8, S9, S10 and S11.

A number of the genes identified in the screen are of unknown function either because they are orthologs of genes with poorly characterized function or because we were unable to identify orthologous genes in the databases and may therefore represent novel *Xenopus* genes. However, analysis of the annotated genes identified in the screen reveals that a large proportion of the genes regulated by FGF signaling, are themselves also involved in gene regulation, either directly, as in the case of transcription factors, or via involvement in other signaling pathways. This indicates the key position of FGFs as upstream regulators of genetic pathways leading to germ layer specification during the late blastula to early gastrula stage of amphibian development. In addition, FGF signaling is required for the normal expression of genes such as *Prickle*, *marginal coil* and *Ephrin receptor A4* (*pagliaccio*) which are involved in regulating cell movements and adhesion during gastrulation [52]–[54].

We also note that the *purine phosphorylase* and *glycogen phosphorylase* genes, which code for enzymes involved in nucleotide and carbohydrate metabolism respectively, are dependent on FGF signaling and are expressed in highly restricted, FGF associated domains in the early mesoderm. Further studies will be required to determine if these genes have previously unsuspected roles in the regulation of developmental mechanisms.

Previous studies have identified a number of FGF inducible inhibitors of the FGF signaling pathway, including the *Sprouty* genes, which we find are significantly down regulated in our screen [36], [55]. In the present study we identify the MAP kinase phosphatase genes *MKP1* and *DUSP5* as FGF targets which are activated rapidly following the mid-blastula transition and show that they are able to inhibit FGF signalling in *Xenopus* tissues. DUSP5 is a novel *Xenopus* MKP which is expressed in the early mesoderm and neural plate in a pattern which is remarkably similar to that of *Xenopus FGF3* [44]. Similar correlation with sites of FGF activity has been reported for *MKP3*, which has also been shown to act as a feedback inhibitor of FGF signaling [34], [56].

The picture that emerges is that activation of FGF signaling induces the production of multiple inhibitors which act to moderate and limit the response to FGF signaling. A number of positive feedback mechanisms also impact on the FGF pathway, including the transcriptional activation the *FLRT3* and *brachyury* genes [6], [57], [58]. FLRT3 is a transmembrane protein that potentiates FGF signal transduction and brachyury is a T-box transcription factor which has been shown to be a component of a positive feedback loop that leads to increased transcription of FGF ligand genes in the early mesoderm [5], [6], [58]. The presence of positive and negative feedback loops which modulate FGF signaling at multiple levels highlights the critical importance for fine tuning the overall levels of FGF signaling during development.

An interesting observation is that expression of the *FoxD3* and *Lin28* genes are down regulated in response to FGF inhibition. The FoxD3 transcription factor is linked to amphibian organizer function [59] but has also been implicated as a component of the pluripotency circuit of mammalian embryonic stem cells via regulation of the *nanog* gene [17], [18]. The *Lin28* gene codes for an RNA binding protein, which together with the *Oct4*, *Sox2* and *Nanog* genes, can convert somatic cells to an embryonic stem cell phenotype [14].

Previous studies have indicated that FGF signaling is required as a competence factor necessary for the response of embryonic amphibian cells to activin-like signals during development [39], [40], [60]. We also note a recent study which showed that FGF signaling in murine ES cells is necessary to allow differentiation into multiple lineages, including mesoderm [12]. These observations raise the intriguing possibility that FGF signaling, acting via downstream targets such as *Lin28* and *FoxD3*, might have a general role in regulating pluripotency or the competence of embryonic cells to respond to signals which direct differentiation both during normal development and in culture.

## Materials and Methods

### Ethics statement

All animal work was undertaken under a licence from the UK Home Office.

### Embryological methods

Embryos were staged according to Nieuwkoop and Faber [61]. Normal embryos were cultured in NAM/10. Injection of embryos with synthetic mRNAs was carried out in 33% NAM+5% ficoll (Sigma) at the 2 or 4-cell stage. Animal caps were explanted from embryos in 50% NAM Recombinant FGF4 [43] treatment was in 50% NAM+ 5 mg/ml BSA.

### Identification of Xenopus tropicalis full length clones

Clones containing full length coding region of *X.tropicalis* MKP1 (DUSP1/XCL100) (accession number AL967533) and DUSP5 (accession number AL648624) were identified using the peptide sequence of *X.laevis* orthologues (accession numbers NM_001088684 and BJ067398 respectively) and BLASTP on the Sanger Institute *X.tropicalis* EST database (www.sanger.ac.uk/Projects/X_tropicalis/).

### mRNA synthesis

Capped mRNA was synthesised using the SP6 Megascript kit (Ambion) and a modified protocol using a 1∶10 ratio of GTP to m7G(5′)Gppp(5′)G cap. All cDNAs used for mRNA were in either pSP64t, Cs2+ or CS107 and were transcribed using SP6 polymerase. The dominant negative *X.laevis* FGFRI (dnFGFR1) plasmid was a gift from Enrique Amaya [24]. The dominant negative *X.laevis* FGFR4a (dnFGFR4) plasmid was a gift from Harumasa Okamoto [25]. The *X.laevis* MKP3 (DUSP6/X17C) plasmid was a gift from Bob Old [62].

### In situ hybridisation

Embryos were fixed in MEMFA and *in situ* hybridizations were carried out as per [63] with the modifications described in [64]. Details for *in situ* probe plasmids are shown in Table S15. The sources of the plasmids, including Geneservice (www.geneservice.co.uk) and the NIBB/NIG/NBRP *X. laevis* EST project (xenopus.nibb.ac.jp) are indicated.

### RNAase protection analysis

RNA extraction and RNAase protection analysis were carried out according to the methods of Pownall et al. (1996). Data relating to RNAase protection plasmids are shown in Table S16.

### Whole-mount immunohistochemistry and Western blotting

dp-ERK immunohistochemistry was carried out according the methods of [22]. Western blot samples were homogenized in PhosphoSafe (Novagen) extraction buffer per embryo. Following centrifugation supernatants were subjected to SDS-PAGE. Gels were blotted onto Immobilon-P (Millipore) transfer membrane. Antibody concentrations were mouse anti-dp-ERK (Sigma), 1∶8000, anti-phosphoSmad1/5/8 (NEB), 1∶500, anti-GAPDH (HyTest), 1∶3000. 1∶8,000, anti-GAPDH (HyTest), 1∶1,000,000, anti-mouse POD (Amersham), 1∶3000 and anti-rabbit POD (Amersham), 1;2000. Peroxidase activity was detected using BM chemiluminescence blotting substrate (Roche) and Hyperfilm (Amersham).

### Embryos for microarray experiments

Embryos were injected with 4 ng of dnFGFR1 or dnFGFR4 mRNA and were collected with sibling controls at early gastrula stage 10.5 (11 hours post-fertilization at 23°C). In order to enable statistical analysis three biological replicates were produced by in vitro fertilization from different pairs of male and female frogs. Each replicate set comprised control, dnFGFR1 injected and dnFGFR4 injected embryos. Before processing for microarray analysis each replicate set was assessed for the effectiveness of FGF signaling inhibition by analysing levels of dp-ERK and levels of the known FGF targets *Xbra*, *Cdx4* and *myoD* expression in sibling embryos.

For the early developmental timecourse embryos from a single mating were cultured at 23°C and collected at 5, 6, 7, 8, 9, 10, 11, 12, 13, 14, 15 and 16 hours post fertilization (NF stage 8, 8.5, 9, 9.5, 10, 10+, 10.5, 11, 12, 12.5, 13 and 14) for microarray analysis. Sibling embryos were also collected at the same time points for Western blot analysis of dp-ERK.

### Preparation of total RNA for microarray analysis

Batches of 10 embryos were extracted in Tri-reagent according manufacturer's protocol (Sigma). RNA was precipitated using isopropanol and cleaned up using the Qiagen RNeasy kit followed by a lithium chloride precipitation. Quality of RNA was assessed using the Agilent 2100 Bioanalyzer.

### Preparation of labelled cRNA and chip hybridization

2 µg of total RNA was processed for the microarray by using the Affymetrix GeneChip one-cycle target labelling kit (Affymetrix) according to the manufacturer's recommended protocols. The quality and quantity of the resulting biotinylated cRNA was determined by using NanoDrop ND 1000 (NanoDrop Technologies) and Agilent Bioanalyzer 2100 (Agilent Technologies). Biotin-labelled cRNA samples were fragmented randomly to 35–200 bp at 94°C in fragmentation buffer (Affymetrix) according to the manufacturer's recommended protocols and aliquots of the fragmented cRNA were run on the Agilent 2100 Bioanalyzer to assess the quality of the generated cRNA.

Biotin-labeled fragmented cRNA samples were combined with hybridization buffer containing hybridization Control cRNA and Control Oligo B2 (Affymetrix), before hybridization to GeneChip® *Xenopus laevis* Genome Array for 16 h at 45°C. The arrays were washed, stained, and scanned using the Affymetrix Model 450 Fluidics Station and Affymetrix Model 3000 scanner using the manufacturer's recommended protocols.

### Microarray data analysis

Affymetrix microarray experiments were conducted in accordance with the MIAME standards requirements [65]. Raw data processing was performed by using the Affymetrix GCOS 1.2 software. After hybridization and scanning, probe cell intensities were calculated and summarized for the respective probe sets by means of the MAS5 algorithm. To compare the expression values of the genes from chip to chip, global scaling was performed, which resulted in the normalization of the trimmed mean of each chip to a target intensity (TGT value) of 500 as per manufacturers documentation. Each sample and hybridization underwent a quality control evaluation checking for adequate scaling factors (1–3 for all samples), percentage of probe sets reliably detected (between 40–60% present call), and optimal 3′/5′ hybridization ratios for the housekeeping genes (e.g., GAPDH), poly(A) spike-in controls, and the prokaryotic controls (bioB, bioC, bioD and cre).

Data were imported into BRB ArrayTools software version 3.6.0 (http://linus.nci.nih.gov/BRB-ArrayTools.html). Imported array data were filtered using the following criteria.

1. **Spot filters-** Threshold minimum value if spot intensity below 5.
2. **Normalization-** Normalize (center) each array using median over entire array.
3. **Exclude a gene under any of the following conditions-** Percent of data missing or filtered out exceeds 50% Percent of absent (i.e., Detection Call = A) data exceeds 50%

Scatterplots were generated using the phenotype averages tool of BRB ArrayTools. Gene lists of FGF targets were generated using the BRB between groups of arrays class comparison tool (unpaired, two sample t-test with random variance model and nominal significance level p = 0.01). An additional filter excluded genes with less than 2-fold difference from controls.

Temporal expression profiles for a given gene were generated in Microsoft Excel by plotting relative expression at each time point as a percentage of the maximum expression level within the time course. Cluster analysis was undertaken using the BRB gene cluster analysis tool (complete linkage and centred correlation). The Affymetrix Cel files for all microarray experiments are available at EMBL ArrayExpress, accession numbers E-MEXP-2058 and E-MEXP-2059.

### Target gene annotation

Target gene annotation was accomplished using a combination of existing Affymetrix Gene array annotation and BLAST searching of target sequences against Genbank, Swiss-prot (www.ncbi.nlm.nih.gov/BLAST/) and NIBB/NIG/NBRP *Xenopus laevis* EST project (xenopus.nibb.ac.jp) databases.

## Supporting Information

Figure S1 Alignment of amphibian and human DUSP5 peptide sequences. Alignment of the peptide sequences for human and Xenopus tropicalis DUSP5 produced by the Clustal W method. Identical residues are boxed in red
(0.15 MB TIF)

Figure S2 Expression of FGF7, FGF10 and FGF20 during amphibian development. In situ hybridisations for showing the expression of FGF7 (A, B and C), FGF10 (D, E and F) and FGF20 (G) at the indicated stages. In situ hybridizations for FGF7 and FGF10 are on Xenopus tropicalis embryos. In situ hybridization for FGF20 is on a Xenopus laevis embryo. (A, C D, E and F) are lateral views with anterior to the left and dorsal to the top. (B) is a posterior view with dorsal to the top. (G) is a vegetal view with dorsal to the top. (A and B) shows expression in the posterior mesoderm and ectoderm (white arrow) around the closed blastopore (bp). Expression is also detected in the anterior endoderm (end). (C) shows expression in the branchial arch region (bra). (D and E) show expression in a domain juxtaposed to the anterior of the otic vesicle (otv, black arrow) and in the branchial arch (bra) region. (F) shows expression in the tailbud (tlb). (G) shows expression in the circumblastoporal region in a distinct dorsal to ventral gradient. The dorsal blastopore lip (dbl) is indicated.
(1.81 MB TIF)

Table S1 Genes differentially regulated in dnFGFR1 versus dnFGFR4 injected embryos
(0.03 MB DOC)

Table S2 Genes positively regulated by FGF signaling involved in transcriptional regulation
(0.07 MB DOC)

Table S3 Genes positively regulated by FGF signaling involved in cell signalling
(0.08 MB DOC)

Table S4 Genes positively regulated by FGF signaling involved in metabolism
(0.04 MB DOC)

Table S5 Genes positively regulated by FGF signaling of other known function
(0.05 MB DOC)

Table S6 Genes positively regulated by FGF signaling of unknown function
(0.04 MB DOC)

Table S7 Genes negatively regulated by FGF signaling involved in transcriptional regulation
(0.03 MB DOC)

Table S8 Genes negatively regulated by FGF signaling involved in cell signalling
(0.03 MB DOC)

Table S9 Genes negatively regulated by FGF signaling involved in metabolism
(0.03 MB DOC)

Table S10 Genes negatively regulated by FGF signaling of other known function
(0.03 MB DOC)

Table S11 Genes negatively regulated by FGF signaling of unknown function
(0.03 MB DOC)

Table S12 GO terms for genes positively regulated by FGF signalling
(0.10 MB DOC)

Table S13 GO terms for genes negatively regulated by FGF signalling
(0.04 MB DOC)

Table S14 Expression of genes positively regulated by FGF signalling
(0.38 MB DOC)

Table S15 In situ clone data
(0.06 MB DOC)

Table S16 RNAase protection probe data
(0.04 MB DOC)
