# Supplementary figures and images for: Characterisation of the Fibroblast Growth Factor Dependent Transcriptome in Early Development
Source: PLoS One. 2009 Mar 31;4(3):e4951. doi: 10.1371/journal.pone.0004951 (PMC2659300; doi:10.1371/journal.pone.0004951)

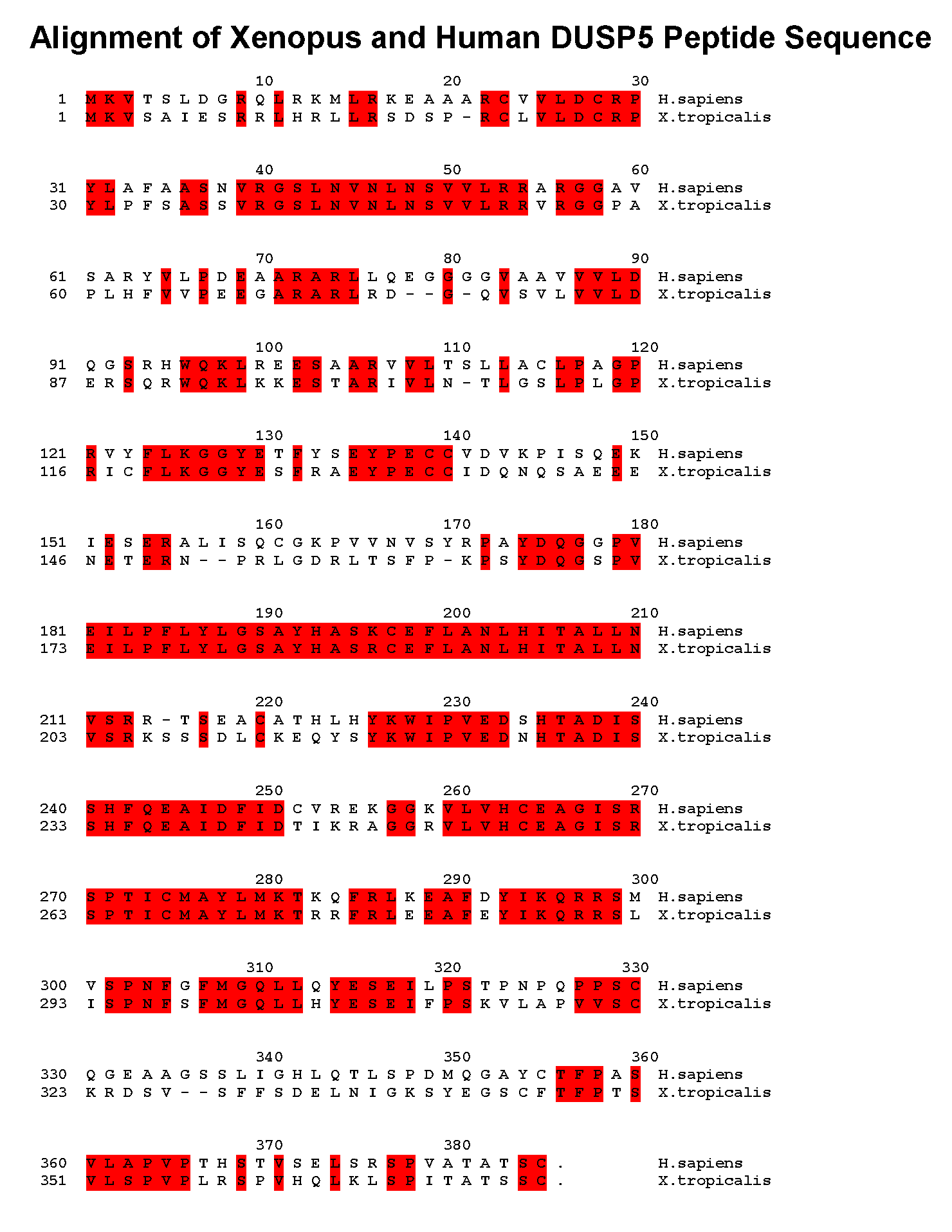

Supplement: Figure S1 — Alignment of amphibian and human DUSP5 peptide sequences. Alignment of the peptide sequences for human and Xenopus tropicalis DUSP5 produced by the Clustal W method. Identical residues are boxed in red (0.15 MB TIF) [file pone.0004951.s001.tif]

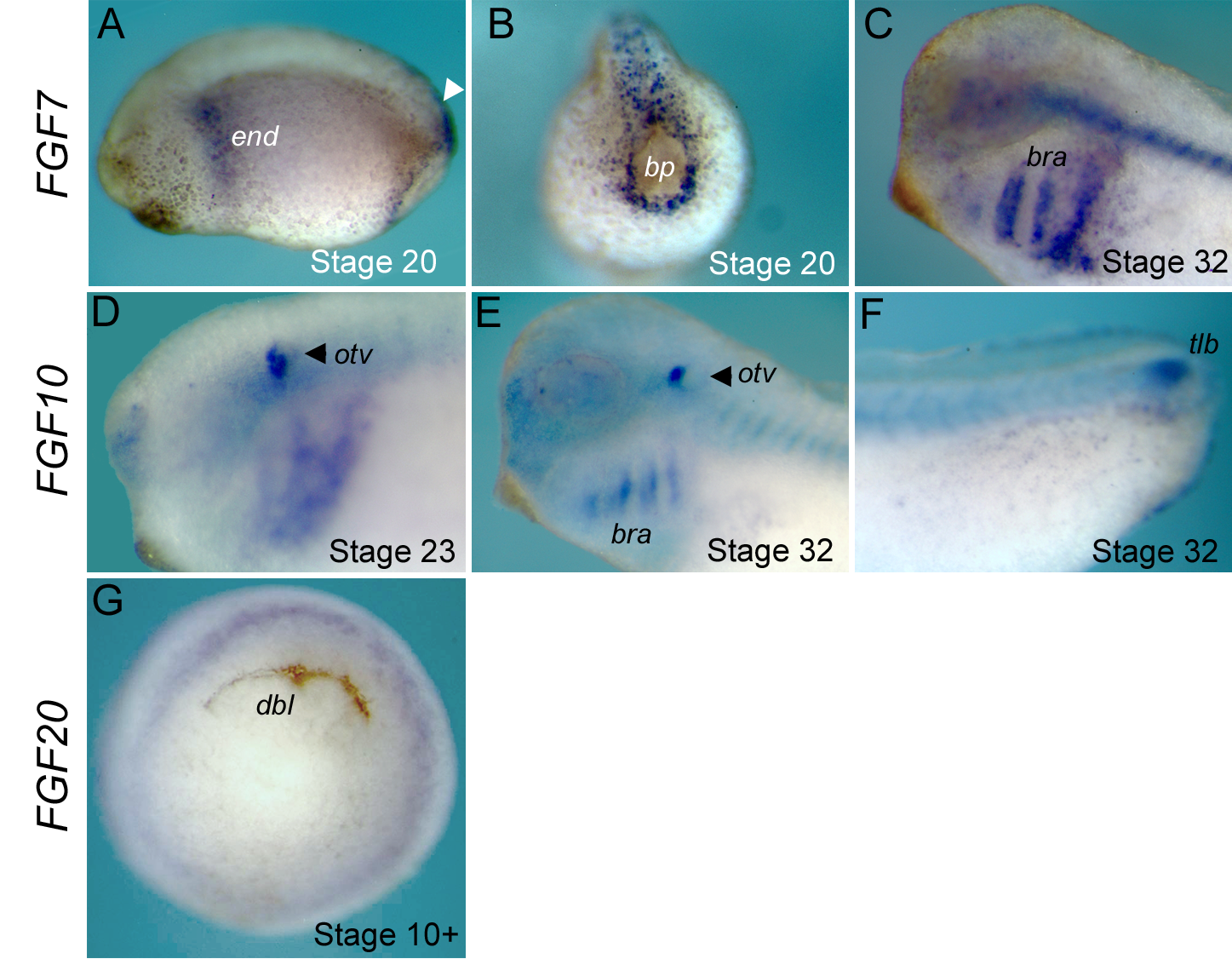

Supplement: Figure S2 — Expression of FGF7, FGF10 and FGF20 during amphibian development. In situ hybridisations for showing the expression of FGF7 (A, B and C), FGF10 (D, E and F) and FGF20 (G) at the indicated stages. In situ hybridizations for FGF7 and FGF10 are on Xenopus tropicalis embryos. In situ hybridization for FGF20 is on a Xenopus laevis embryo. (A, C D, E and F) are lateral views with anterior to the left and dorsal to the top. (B) is a posterior view with dorsal to the top. (G) is a vegetal view with dorsal to the top. (A and B) shows expression in the posterior mesoderm and ectoderm (white arrow) around the closed blastopore (bp). Expression is also detected in the anterior endoderm (end). (C) shows expression in the branchial arch region (bra). (D and E) show expression in a domain juxtaposed to the anterior of the otic vesicle (otv, black arrow) and in the branchial arch (bra) region. (F) shows expression in the tailbud (tlb). (G) shows expression in the circumblastoporal region in a distinct dorsal to ventral gradient. The dorsal blastopore lip (dbl) is indicated. (1.81 MB TIF) [file pone.0004951.s002.tif]
