# Supplementary material for: Characterisation of the Fibroblast Growth Factor Dependent Transcriptome in Early Development
Source: PLoS One. 2009 Mar 31;4(3):e4951. doi: 10.1371/journal.pone.0004951 (PMC2659300; doi:10.1371/journal.pone.0004951)
Supplement: Table S1 — Genes differentially regulated in dnFGFR1 versus dnFGFR4 injected embryos (0.03 MB DOC) [file pone.0004951.s003.doc]

**Table S1 Genes differentially regulated in dnFGFR1 versus dnFGFR4 injected embryos**

| **Gene** | **Fold difference (dnFGFR4/dnFGFR1)** | **GenBank**  **accession** | **Affymetrix**  **probe set** |
| --- | --- | --- | --- |
| Cdx4 | 2.8 | BJ05440 | Xl.16733.1.A1_at |
| Connexin 29 | 2.6 | BJ076720 | Xl.8924.1.A1_at |
| Frizzled 10A | 2.4 | AB0456534 | Xl.1040.1.S1_at |
| Twik-2 | 2.0 | BJ048106 | Xl.23586.1.A1_at |
| XSpr2 | 2.0 | AY062263 | Xl.2755.2.A1_at |
