# Supplementary material for: Characterisation of the Fibroblast Growth Factor Dependent Transcriptome in Early Development
Source: PLoS One. 2009 Mar 31;4(3):e4951. doi: 10.1371/journal.pone.0004951 (PMC2659300; doi:10.1371/journal.pone.0004951)
Supplement: Table S2 — Genes positively regulated by FGF signaling involved in transcriptional regulation (0.07 MB DOC) [file pone.0004951.s004.doc]

**Table S2 Genes positively regulated by FGF signaling involved in transcriptional regulation**

| **Gene** | **Notes** |
| --- | --- |
| Brachyury | Known FGF target.T-box transcription factor involved in regulating properties of the mesoderm [1]. |
| Cdx1 | Known FGF target. Homeodomain transcription factor required in posterior development [2]. |
| Cdx4 | Known FGF target. Homeodomain transcription factor required for posterior development [3]. |
| Egr1 | Known MAP kinase target. Zinc finger transcription factor expressed in organizer region [4]. |
| Esr5 | bHLH transcription factor [5]. |
| FoxA4a | Forkhead domain transcription factor (Pintallavis) expressed in organizer and dorsal midline [6] |
| FoxC1 | Forkhead domain transcription factor (XFD-11) expressed in ventrolateral mesoderm [7]. |
| FoxD3A | Forkhead domain transcription factor expressed in the organizer region [8]. |
| FoxD5A | Forkhead domain transcription factor expressed in the organizer region [9]. |
| Goosecoid | Homeodomain transcription factor expressed in the organizer region [10]. |
| Oct1 | POU domain transcription factor [11]. |
| SIP1 | Zinc finger protein [12]. |
| Xiro3 | Known FGF target. Homeodomain transcription factor expressed in the organizer region [13]. |
| Xom | Homeodomain transcription factor (vent2) expressed in ventro-lateral mesoderm [14]. |
| XSpr2 | SP5 related Zinc finger transcription factor [15]. |
| Zic3a | Odd-paired related zinc finger transcription factor [16]. |

**References**

1. Isaacs HV, Pownall ME, Slack JMW (1994) eFGF regulates Xbra expression during Xenopus gastrulation. embo journal 13: 4469-4481.

2. Keenan ID, Sharrard RM, Isaacs HV (2006) FGF signal transduction and the regulation of Cdx gene expression. developmental biology 299: 478-488.

3. Pownall ME, Tucker AS, Slack JMW, Isaacs HV (1996) eFGF, Xcad3 and Hox genes form a molecular pathway that establishes the anteroposterior axis in Xenopus. development 122: 3881-3892.

4. Panitz F, Krain B, Hollemann T, Nordheim A, Pieler T (1998) The Spemann organizer-expressed zinc finger gene Xegr-1 responds to the MAP kinase/Ets-SRF signal transduction pathway. embo journal 17: 4414-4425.

5. Sparrow DB, Jen WC, Kotecha S, Towers N, Kintner C, et al. (1998) Thylacine 1 is expressed segmentally within the paraxial mesoderm of the Xenopus embryo and interacts with the Notch pathway. development 125: 2041-2051.

6. Ruiz i Altaba A, Jessell TM (1992) Pintallavis, a gene expressed in the organizer and midline cells of frog embryos: Involvement in the development of the neural axis. development 116: 81-93.

7. Köster M, Dillinger K, Knöchel W (1998) Expression pattern of the winged helix factor XFD-11 during Xenopus embryogenesis. mechanisms of development 76: 169-173.

8. Steiner AB, Engleka MJ, Lu Q, Piwarzyk EC, Yaklichkin S, et al. (2006) FoxD3 regulation of Nodal in the Spemann organizer is essential for Xenopus dorsal mesoderm development. development 133: 4827-4838.

9. Sullivan SA, Akers L, Moody SA (2001) foxD5a, a Xenopus winged helix gene, maintains an immature neural ectoderm via transcriptional repression that is dependent on the C- terminal domain. developmental biology 232: 439-457.

10. Cho KWY, Blumberg B, Steinbeisser H, De Robertis EM (1991) Molecular nature of Spemann's Organizer: the role of the *Xenopus* homeobox gene *goosecoid*. cell 67: 1111-1120.

11. Veenstra G, Beumer T, PetersonMaduro J, Stegeman B, Karg H, et al. (1995) Dynamic and differential Oct-1 expression during early xenopus embryogenesis: Persistence of Oct-1 protein following down-regulation of the RNA. mechanisms of development 50: 103-117.

12. Nitta KR, Tanegashima K, Takahashi S, Asashima M (2004) XSIP1 is essential for early neural gene expression and neural differentiation by suppression of BMP signaling. developmental biology 275: 258-267.

13. Bellefroid EJ, Kobbe A, Gruss P, Pieler T, Gurdon JB, et al. (1998) Xiro3 encodes a Xenopus homolog of the Drosophila Iroquois genes and functions in neural specification. embo journal 17: 191-203.

14. Ladher R, Mohun TJ, Smith JC, Snape AM (1996) Xom: a Xenopus homeobox gene that mediates the early effects of BMP-4. development 122: 2385-2394.

15. Ossipova O, Stick R, Pieler T (2002) XSPR-1 and XSPR-2, novel Sp1 related zinc finger containing genes, are dynamically expressed during Xenopus embryogenesis. mechanisms of development 115: 117-122.

16. Nakata K, Nagai T, Aruga J, Mikoshiba K (1997) Xenopus Zic3, a primary regulator both in neural and neural crest development. proceedings of the national academy of sciences of the united states of america 94: 11980-11985.
