# Supplementary material for: Characterisation of the Fibroblast Growth Factor Dependent Transcriptome in Early Development
Source: PLoS One. 2009 Mar 31;4(3):e4951. doi: 10.1371/journal.pone.0004951 (PMC2659300; doi:10.1371/journal.pone.0004951)
Supplement: Table S3 — Genes positively regulated by FGF signaling involved in cell signalling (0.08 MB DOC) [file pone.0004951.s005.doc]

**Table S3 Genes positively regulated by FGF signaling involved in cell signalling**

| **Gene** | **Notes** |
| --- | --- |
| ADMP | BMP-like factor expressed in the organizer region [1]. |
| Chordin | Secreted BMP inhibitor expressed in the organizer region [2]. |
| Crescent | Frizzled domain containing protein expressed in organizer region [3]. |
| Dkk1 | Wnt inhibitor expressed in the organizer region [4]. |
| DUSP5 | Novel MAP kinase phosphatase |
| Ephrin receptor A2 | Receptor tyrosine kinase [5]. |
| Ephrin receptor A4 | Pagliaccio tyrosine kinase receptor [6]. |
| Frzb1 | Secreted Wnt inhibitor [7]. |
| Connexin 29 | Putative identification |
| G-coupled receptor P2Y5 | Putative LPA receptor [8]. |
| Gravin-like | Related to A-kinase anchoring proteins [9]. |
| Grb10 interacting protein2 | Putative identification. Similar to grb10 adaptor protein |
| MKP1 | Xcl100 map kinase phosphatase [10]. |
| Noggin | Secreted BMP antagonist [11]. |
| p75-like fullback receptor | Fullback neurotrophin receptor homolog [12]. |
| Prickle | Component of planar cell polarity signalling pathway [13]. |
| RALDH2 | Enzyme involved in retinoic acid metabolism [14]. |
| Related to DC-STAMP domain receptor | Putative identification. |
| Sprouty1 | Known target and inhibitor of the FGF signalling pathway [15]. |
| Sprouty2 | Known target and inhibitor of the FGF signalling pathway [15]. |
| Wnt5b | Wnt5b homolog |
| Wnt8 | Canonical Wnt ligand [16]. |

**References**

1. Moos Jr M, Wang S, Krinks M (1995) Anti-Dorsalizing Morphogenetic Protein is a novel TGF-beta homolog expressed in the Spemann organizer. development 121: 4293-4301.

2. Holley SA, Jackson PD, Sasai Y, Lu B, De Robertis EM, et al. (1995) A conserved system for dorsal-ventral patterning in insects and vertebrates involving sog and chordin. nature 376: 249-253.

3. Shibata M, Ono H, Hikasa H, Shinga J, Taira M (2000) Xenopus crescent encoding a Frizzled-like domain is expressed in the Spemann organizer and pronephros. mechanisms of development 96: 243-246.

4. Glinka A, Wu W, Delius H, Monaghan AP, Blumenstock C, et al. (1998) Dickkopf-1 is a member of a new family of secreted proteins and functions in head induction. nature 391: 357-362.

5. Helbling PM, Tran CT, Brändli AW (1998) Requirement for EphA receptor signaling in the segregation of Xenopus third and fourth arch neural crest cells. mechanisms of development 78: 63-79.

6. Scales J, Winning R, Renaud C, Shea L, Sargent T (1995) Novel members of the eph receptor tyrosine kinase subfamily expressed during Xenopus development. oncogene 11: 1745-1752.

7. Wang S, Krinks M, Lin K, Luyten FP, Moos M, Jr. (1997) Frzb, a secreted protein expressed in the Spemann organizer, binds and inhibits Wnt-8. cell 88: 757-766.

8. Pasternack SM, von Kugelgen I, Aboud KA, Lee YA, Ruschendorf F, et al. (2008) G protein-coupled receptor P2Y5 and its ligand LPA are involved in maintenance of human hair growth. nature genetics 40: 329-334.

9. Klingbeil P, Frazzetto G, Bouwmeester T (2001) Xgravin-like (Xgl), a novel putative a-kinase anchoring protein (AKAP) expressed during embryonic development in Xenopus. mechanisms of development 100: 323-326.

10. Lewis T, Groom LA, Sneddon AA, Smythe C, Keyse SM (1995) XCL100, an inducible nuclear MAP kinase phosphatase from Xenopus laevis: its role in MAP kinase inactivation in differentiated cells and its expression during early development. journal of cell science 108 ( Pt 8): 2885-2896.

11. Smith WC, Harland RM (1992) Expression cloning of noggin, a new dorsalizing factor localized to the Spemann organizer in Xenopus embryos. cell 70: 829-840.

12. Bromley E, Knapp D, Wardle FC, Sun BI, Collins-Racie L, et al. (2004) Identification and characterisation of the posteriorly-expressed Xenopus neurotrophin receptor homolog genes fullback and fullback-like. Gene Expr Patterns 5: 135-140.

13. Wallingford JB, Goto T, Keller R, Harland RM (2002) Cloning and expression of Xenopus Prickle, an orthologue of a Drosophila planar cell polarity gene. mechanisms of development 116: 183-186.

14. Chen Y, Pollet N, Niehrs C, Pieler T (2001) Increased XRALDH2 activity has a posteriorizing effect on the central nervous system of Xenopus embryos. mechanisms of development 101: 91-103.

15. Nutt SL, Dingwell KS, Holt CE, Amaya E (2001) Xenopus Sprouty2 inhibits FGF-mediated gastrulation movements but does not affect mesoderm induction and patterning. genes and development 15: 1152-1166.

16. Smith WC, Harland RM (1991) Injected Xwnt-8 RNA acts early in Xenopus embryos to promote formation of a vegetal dorsalizing center. cell 67: 753-765.
