# Supplementary material for: Characterisation of the Fibroblast Growth Factor Dependent Transcriptome in Early Development
Source: PLoS One. 2009 Mar 31;4(3):e4951. doi: 10.1371/journal.pone.0004951 (PMC2659300; doi:10.1371/journal.pone.0004951)
Supplement: Table S4 — Genes positively regulated by FGF signaling involved in metabolism (0.04 MB DOC) [file pone.0004951.s006.doc]

**Table S4 Genes positively regulated by FGF signaling involved in metabolism**

| **Gene** | **Notes** |
| --- | --- |
| Alkaline phosphatase | Xenopus alkaline phosphatase [1]. |
| Fructokinase-related protein | Putative identification |
| Glut1 transporter | Glucose transporter [2]. |
| Glycogen phophorylase | Enzyme involved in breakdown of glycogen |
| NADH dehydrogenase sub-unit | Known FGF target [3]. |
| Purine phosphorylase | Enzyme involved in nucleotide metabolism |

**References**

1. Klein SL, Strausberg RL, Wagner L, Pontius J, Clifton SW, et al. (2002) Genetic and genomic tools for Xenopus research: The NIH Xenopus initiative. developmental dynamics 225: 384-391.

2. Suzawa K, Yukita A, Hayata T, Goto T, Danno H, et al. (2007) Xenopus glucose transporter 1 (xGLUT1) is required for gastrulation movement in Xenopus laevis. international journal of developmental biology 51: 183-190.

3. Chung HA, Hyodo-Miura J, Kitayama A, Terasaka C, Nagamune T, et al. (2004) Screening of FGF target genes in Xenopus by microarray: temporal dissection of the signalling pathway using a chemical inhibitor. Genes Cells 9: 749-761.
