# Supplementary material for: Characterisation of the Fibroblast Growth Factor Dependent Transcriptome in Early Development
Source: PLoS One. 2009 Mar 31;4(3):e4951. doi: 10.1371/journal.pone.0004951 (PMC2659300; doi:10.1371/journal.pone.0004951)
Supplement: Table S5 — Genes positively regulated by FGF signaling of other known function (0.05 MB DOC) [file pone.0004951.s007.doc]

**Table S5 Genes positively regulated by FGF signaling of other known function**

| **Gene** | **Notes** |
| --- | --- |
| Apobec-2 | Orthologue of Apobec-2 RNA editing enzyme [1]. |
| Cytochrome B561 | Involved in electron transport chain [2] |
| Lin28a | RNA binding protein [3] |
| Marginal coil | Known FGF target required for normal morphogenesis [4]. |
| Mitogenic phosphoprotein 67 | [5] |
| Paraxial protocadherin | Sprouty antagonist. Regulator of planar cell polarity signalling [6]. |
| Pinhead | Novel protein involved in head development [7]. |
| Putative methyltransferase | Putative identification. SAM methylase family related enzyme |
| Retrotransposon protein 1A11 | Known FGF target [8]. |
| SMCT | Putative sodium monocarboxylate transporter |

**References**

1. Liao W, Hong SH, Chan BH, Rudolph FB, Clark SC, et al. (1999) APOBEC-2, a cardiac- and skeletal muscle-specific member of the cytidine deaminase supergene family. biochemical and biophysical research communications 260: 398-404.

2. Srivastava M (1996) Xenopus cytochrome b561: molecular confirmation of a general five transmembrane structure and developmental regulation at the gastrula stage. dna and cell biology 15: 1075-1080.

3. Moss EG, Tang L (2003) Conservation of the heterochronic regulator Lin-28, its developmental expression and microRNA complementary sites. developmental biology 258: 432-442.

4. Frazzetto G, Klingbeil P, Bouwmeester T (2002) Xenopus marginal coil (Xmc), a novel FGF inducible cytosolic coiled-coil protein regulating gastrulation movements. mechanisms of development 113: 3-14.

5. Georgi AB, Stukenberg PT, Kirschner MW (2002) Timing of events in mitosis. current biology 12: 105-114.

6. Wang Y, Janicki P, Koster I, Berger CD, Wenzl C, et al. (2008) Xenopus Paraxial Protocadherin regulates morphogenesis by antagonizing Sprouty. genes and development 22: 878-883.

7. Kenwrick S, Amaya E, Papalopulu N (2004) Pilot morpholino screen in Xenopus tropicalis identifies a novel gene involved in head development. developmental dynamics 229: 289-299.

8. Greene JM, Otani H, Good PJ, Dawid IB (1993) A novel family of retrotransposon-like elements in Xenopus laevis with a transcript inducible by two growth factors. nucleic acids research 21: 2375-2381.
