# Supplementary material for: Characterisation of the Fibroblast Growth Factor Dependent Transcriptome in Early Development
Source: PLoS One. 2009 Mar 31;4(3):e4951. doi: 10.1371/journal.pone.0004951 (PMC2659300; doi:10.1371/journal.pone.0004951)
Supplement: Table S6 — Genes positively regulated by FGF signaling of unknown function (0.04 MB DOC) [file pone.0004951.s008.doc]

**Table S6 Genes positively regulated by FGF signaling of unknown function**

| **Accession number** | **Affymetrix**  **probe set** | **Notes** |
| --- | --- | --- |
| AW460550 | Xl.11594.1.A1_at |  |
| BF615090 | Xl.7720.1.A1_at | Meso5. No open reading frame detected [1]. |
| BG555868 | Xl.3023.1.A1_at | Rexp52 [1]. |
| BI312705 | Xl.18179.1.S1_at |  |
| BJ056692 | Xl.15382.1.A1_at |  |
| BJ079872 | Xl.3365.1.A1_at | Weakly similar to Rab1 |
| BJ085271 | Xl.1521.1.A1_at |  |
| BJ088835 | Xl.15887.1.S1_x_at | Similar to Brain protein 44 |
| BJ092401 | Xl.5479.1.A1_at |  |
| BM179370 | Xl.14776.1.A1_at | Putative nucleolar binding protein |
| CA972457 | Xl.19961.1.S1_at |  |
| CB756627 | Xl.25136.1.A1_at | Similar to uncharacterised protein C2orf32 |

**References**

1. Baldessari D, Shin Y, Krebs O, Konig R, Koide T, et al. (2005) Global gene expression profiling and cluster analysis in Xenopus laevis. mechanisms of development 122: 441-475.
