# Supplementary material for: Characterisation of the Fibroblast Growth Factor Dependent Transcriptome in Early Development
Source: PLoS One. 2009 Mar 31;4(3):e4951. doi: 10.1371/journal.pone.0004951 (PMC2659300; doi:10.1371/journal.pone.0004951)
Supplement: Table S7 — Genes negatively regulated by FGF signaling involved in transcriptional regulation (0.03 MB DOC) [file pone.0004951.s009.doc]

**Table S7 Genes negatively regulated by FGF signaling involved in transcriptional regulation**

| **Gene** | **Notes** |
| --- | --- |
| CP2-like transcription factor | Similar to X.tropicalis grainyhead like-2 transcription factor [1]. |
| Glucocorticoid inducible leucine zipper | Tsc22 related transcription factor [1] |
| Hes-related 1b | bHLH transcription factor [2] |
| XANF1 | Homeodomain transcription factor expressed in anterior neural plate [3]. |

**References**

1. Klein SL, Strausberg RL, Wagner L, Pontius J, Clifton SW, et al. (2002) Genetic and genomic tools for Xenopus research: The NIH Xenopus initiative. developmental dynamics 225: 384-391.

2. Shinga J, Itoh M, Shiokawa K, Taira S, Taira M (2001) Early patterning of the prospective midbrain-hindbrain boundary by the HES-related gene XHR1 in Xenopus embryos. mechanisms of development 109: 225-239.

3. Zaraisky A, Lukyanov S, Vasiliev O, Smirnov Y, Belyavsky A, et al. (1992) A novel homeobox gene expressed in the anterior neural plate of the Xenopus embryo. developmental biology 152: 373-382.
