# Supplementary material for: Characterisation of the Fibroblast Growth Factor Dependent Transcriptome in Early Development
Source: PLoS One. 2009 Mar 31;4(3):e4951. doi: 10.1371/journal.pone.0004951 (PMC2659300; doi:10.1371/journal.pone.0004951)
Supplement: Table S8 — Genes negatively regulated by FGF signaling involved in cell signalling (0.03 MB DOC) [file pone.0004951.s010.doc]

**Table S8 Genes negatively regulated by FGF signaling involved in cell signalling**

| **Gene** | **Notes** |
| --- | --- |
| PDGF A chain | [1] |

**References**

1. Bejcek BE, Li DY, Deuel TF (1990) Nucleotide sequence of a cDNA clone of Xenopus platelet-derived growth factor A-chain. nucleic acids research 18: 680.
