# Supplementary material for: Characterisation of the Fibroblast Growth Factor Dependent Transcriptome in Early Development
Source: PLoS One. 2009 Mar 31;4(3):e4951. doi: 10.1371/journal.pone.0004951 (PMC2659300; doi:10.1371/journal.pone.0004951)
Supplement: Table S9 — Genes negatively regulated by FGF signaling involved in metabolism (0.03 MB DOC) [file pone.0004951.s011.doc]

**Table S9 Genes negatively regulated by FGF signaling involved in metabolism**

| **Gene** | **Notes** |
| --- | --- |
| Adenosine deaminase | Putative identification |
| Darmin | Glutamate decarboxypeptidase-like protein expressed in developing endoderm [1] |
| ODC2 | Enzyme involved in polyamine synthesis. Restricted expression along body axis [2] |
| Selenophosphate synthetase 1 | Putative identification |
| Thioredoxin binding protein 2 | Putative identification |

**References**

1. Pera EM, Martinez SL, Flanagan JJ, Brechner M, Wessely O, et al. (2003) Darmin is a novel secreted protein expressed during endoderm development in Xenopus. Gene Expr Patterns 3: 147-152.

2. Cao Y, Zhao H, Hollemann T, Chen Y, Grunz H (2001) Tissue-specific expression of an Ornithine decarboxylase paralogue, XODC2, in Xenopus laevis. mechanisms of development 102: 243-246.
