# Supplementary material for: Characterisation of the Fibroblast Growth Factor Dependent Transcriptome in Early Development
Source: PLoS One. 2009 Mar 31;4(3):e4951. doi: 10.1371/journal.pone.0004951 (PMC2659300; doi:10.1371/journal.pone.0004951)
Supplement: Table S10 — Genes negatively regulated by FGF signaling of other known function (0.03 MB DOC) [file pone.0004951.s012.doc]

**Table S10 Genes negatively regulated by FGF signaling of other known function**

| **Gene** | **Notes** |
| --- | --- |
| WIG | Putative transmembrane protein TA-2 induced in response to wounding [1]. |
| WIG-related | Similar to transmembrane protein TA-2 |
| XIRG protein | Marker of non-neural ectoderm [2]. |

**References**

1. Klingbeil P, Frazzetto G, Bouwmeester T (2001) Xwig1, a novel putative endoplasmic reticulum protein expressed during epithelial morphogenesis and in response to embryonic wounding. international journal of developmental biology 45: 379-385.

2. Schmidt G, Richter K (2000) Expression pattern of XIRG, a marker for non-neural ectoderm. dev genes evol 210: 575-578.
