# Supplementary material for: Characterisation of the Fibroblast Growth Factor Dependent Transcriptome in Early Development
Source: PLoS One. 2009 Mar 31;4(3):e4951. doi: 10.1371/journal.pone.0004951 (PMC2659300; doi:10.1371/journal.pone.0004951)
Supplement: Table S11 — Genes negatively regulated by FGF signaling of unknown function (0.03 MB DOC) [file pone.0004951.s013.doc]

**Table S11 Genes negatively regulated by FGF signaling of unknown function**

| **Accession number** | **Affymetrix**  **probe set** | **Notes** |
| --- | --- | --- |
| AW147865 | Xl.2077.1.A1_at |  |
| AW460608 | Xl.11598.1.A1_at |  |
| BM192746 | Xl.25985.1.A1_at |  |
