# Supplementary material for: Characterisation of the Fibroblast Growth Factor Dependent Transcriptome in Early Development
Source: PLoS One. 2009 Mar 31;4(3):e4951. doi: 10.1371/journal.pone.0004951 (PMC2659300; doi:10.1371/journal.pone.0004951)
Supplement: Table S12 — GO terms for genes positively regulated by FGF signalling (0.10 MB DOC) [file pone.0004951.s014.doc]

**Table S12** GO terms for genes positively regulated by FGF signalling

| **Gene** | **Affymetrix probe set** | **GO terms** |
| --- | --- | --- |
| Brachyury | Xl.514.1.S1_at | biological process|transcription|IEA|GO:0006350//biological process|regulation of transcription, DNA-dependent|IEA|GO:0006355//biological process|development|IEA|GO:0007275//cellular component|nucleus|IEA|GO:0005634//molecular function|DNA binding|IEA|GO:0003677//molecular function|transcription factor activity|IEA|GO:0003700 |
| Egr1 | Xl.637.1.A1_at | cellular component|intracellular|IEA|GO:0005622//molecular function|nucleic acid binding|IEA|GO:0003676//molecular function|zinc ion binding|IEA|GO:0008270//molecular function|metal ion binding|IEA|GO:0046872 |
| FoxD5A | Xl.642.1.S1_at | biological process|regulation of transcription, DNA-dependent|IEA|GO:0006355//cellular component|nucleus|IEA|GO:0005634//molecular function|DNA binding|IEA|GO:0003677//molecular function|transcription factor activity|IEA|GO:0003700//molecular function|sequence-specific DNA binding|IEA|GO:0043565 |
| SIP1 | Xl.958.1.S2_at | biological process|regulation of transcription, DNA-dependent|IEA|GO:0006355//biological process|regulation of transcription|IEA|GO:0045449//cellular component|intracellular|IEA|GO:0005622//cellular component|nucleus|IEA|GO:0005634 |
| Cdx4 | Xl.10269.1.S1_at | biological process|regulation of transcription, DNA-dependent|IEA|GO:0006355//biological process|development|IEA|GO:0007275//biological process|regulation of transcription|IEA|GO:0045449//cellular component|nucleus|IEA|GO:0005634//molecular function|DNA binding|IEA|GO:0003677//molecular function|transcription factor activity|IEA|GO:0003700//molecular function|sequence-specific DNA binding|IEA|GO:0043565 |
| Esr5 | Xl.14524.1.S1_at | biological process|regulation of transcription, DNA-dependent|IEA|GO:0006355//biological process|regulation of transcription|IEA|GO:0045449//cellular component|nucleus|IEA|GO:0005634//molecular function|DNA binding|IEA|GO:0003677//molecular function|transcription regulator activity|IEA|GO:0030528 |
| Purine phosphorylase | Xl.16206.1.A1_at | biological process|nucleobase, nucleoside, nucleotide and nucleic acid metabolism|IEA|GO:0006139 |
| Marginal coil | Xl.5454.1.S1_at | None |
| Paraxial protocadherin | Xl.6173.1.A1_at | biological process|homophilic cell adhesion|IEA|GO:0007156//cellular component|membrane|IEA|GO:0016020//molecular function|calcium ion binding|IEA|GO:0005509 |
| Glycogen phosphorylase | Xl.7815.1.A1_at | biological process|carbohydrate metabolism|IEA|GO:0005975//molecular function|phosphorylase activity|IEA|GO:0004645//molecular function|pyridoxal phosphate binding|IEA|GO:0030170 |
| NADH dehydrogenase sub-unit | Xl.12993.1.A1_at | None |
| FoxD3A | Xl.525.1.S1_at | biological process|regulation of transcription, DNA-dependent|IEA|GO:0006355//cellular component|nucleus|IEA|GO:0005634//molecular function|DNA binding|IEA|GO:0003677//molecular function|transcription factor activity|IEA|GO:0003700//molecular function|sequence-specific DNA binding|IEA|GO:0043565 |
| G-coupled receptor P2Y5 | Xl.19933.1.S1_at | None |
| Related to DC-STAMP domain receptor | Xl.15270.1.A1_at | None |
| Meso05 | Xl.7720.1.A1_at | None |
| Uncharacterised protein C2orf32 | Xl.25136.1.A1_at | None |
| Frzb1 | Xl.212.2.S1_a_at | cellular component|extracellular region|IEA|GO:0005576//molecular function|protein binding|IEA|GO:0005515 |
| XPO | Xl.5908.1.S1_s_at | biological process|multicellular organismal development|IEA|GO:0007275 |
| Ephrin receptor A4 | Xl.13.2.A1_at | biological process|protein amino acid phosphorylation|IEA|GO:0006468//biological process|transmembrane receptor protein tyrosine kinase signaling pathway|IEA|GO:0007169//cellular component|membrane|IEA|GO:0016020//cellular component|integral to membrane|IEA|GO:0016021 |
| XSpr2 | Xl.2755.1.S1_a_at | cellular component|intracellular|IEA|GO:0005622//molecular function|nucleic acid binding|IEA|GO:0003676//molecular function|zinc ion binding|IEA|GO:0008270 |
| Zic3a | Xl.7969.1.S1_at | cellular component|intracellular|IEA|GO:0005622//cellular component|nucleus|IEA|GO:0005634 |
| Xiro3 | Xl.4522.1.S1_at | biological process|regulation of transcription, DNA-dependent|IEA|GO:0006355//biological process|regulation of transcription|IEA|GO:0045449//cellular component|nucleus|IEA|GO:0005634//molecular function|DNA binding|IEA|GO:0003677//molecular function|transcription factor activity|IEA|GO:0003700//molecular function|sequence-specific DNA binding|IEA|GO:0043565 |
| Gravin-like | Xl.3468.1.S1_at | biological process|protein targeting|IEA|GO:0006605//biological process|signal transduction|IEA|GO:0007165//molecular function|protein binding|IEA|GO:0005515 |
| Alkaline phosphatase | Xl.1299.1.S1_at | biological process|metabolic process|IEA|GO:0008152 |
| Apobec2 | Xl.5876.1.A1_a | molecular function|zinc ion binding|IEA|GO:0008270//molecular function|hydrolase activity, acting on carbon-nitrogen (but not peptide) bonds, in cyclic amidines|IEA|GO:0016814 |
| p75-like fullback receptor | Xl.3540.1.S1_at | biological process|signal transduction|IEA|GO:0007165//cellular component|integral to membrane|IEA|GO:0016021//molecular function|receptor activity|IEA|GO:0004872//molecular function|protein binding|IEA|GO:0005515 |
| Wnt8 | Xl.49.1.S1_at | cellular component|extracellular region|IEA|GO:0005576//cellular component|proteinaceous extracellular matrix|IEA|GO:0005578//molecular function|signal transducer activity|IEA|GO:0004871//molecular function|protein binding|IPI|GO:0005515 |
| Fructokinase-related protein | Xl.15623.1.A1_at | biological process|fructose 2,6-bisphosphate metabolic process|IEA|GO:0006003//molecular function|catalytic activity|IEA|GO:0003824//molecular function|ATP binding|IEA|GO:0005524 |
| Crescent | Xl.619.1.S1_at | cellular component|extracellular region|IEA|GO:0005576//molecular function|protein binding|IEA|GO:0005515 |
| Pinhead | Xl.3529.1.A1_at | None |
| Wnt5b | Xl.11619.1.S1_at | None |
| Unknown | Xl.5479.1.A1_at | None |
| Retrotransposon protein 1a11 | Xl.3352.1.S1_at | biological process|regulation of transcription, DNA-dependent|IEA|GO:0006355//biological process|proteolysis|IEA|GO:0006508//cellular component|nucleus|IEA|GO:0005634 |
| FoxA4 | Xl.1082.1.S1_at | cellular component|nucleus|IEA|GO:0005634//cellular component|nucleus|NAS|GO:0005634 |
| Mitotic phosphoprotein 67 | Xl.20772.1.A1_at | None |
| Cdx1 | Xl.23739.1.A1_at | cellular component|nucleus|IEA|GO:0005634//molecular function|DNA binding|IEA|GO:0003677//molecular function|transcription factor activity|IEA|GO:0003700//molecular function|sequence-specific DNA binding|IEA|GO:0043565 |
| Sprouty2 | Xl.11965.1.S1_at | biological process|multicellular organismal development|IEA|GO:0007275//biological process|regulation of signal transduction|IEA|GO:0009966//cellular component|membrane|IEA|GO:0016020 |
| DUSP5 | Xl.15374.1.A1_at | None |
| Chordin | Xl.3549.1.S1_at | cellular component|extracellular region|IEA|GO:0005576//cellular component|extracellular region|NAS|GO:0005576//molecular function|heparin binding|ISS|GO:0008201//molecular function|syndecan binding|ISS|GO:0045545 |
| MKP1 | Xl.2803.1.S1_at | biological process|protein amino acid dephosphorylation|IEA|GO:0006470//biological process|dephosphorylation|IEA|GO:0016311 |
| Unknown | Xl.18179.1.S1_at | None |
| Xom | Xl.37.1.S1_at | biological process|regulation of transcription, DNA-dependent|IEA|GO:0006355//biological process|regulation of transcription|IEA|GO:0045449//cellular component|nucleus|IEA|GO:0005634//molecular function|DNA binding|IEA|GO:0003677//molecular function|transcription factor activity|IEA|GO:0003700//molecular function|sequence-specific DNA binding|IEA|GO:0043565 |
| Putative nucleolar GTP binding protein | Xl.14776.1.A1_at | None |
| Lin28a homologue | Xl.3418.1.A1_at | None |
| Glut1 transporter | Xl.24121.1.A1_at | None |
| Unknown | Xl.15382.1.A1_at | None |
| Dkk1 | Xl.251.1.S1_at | biological process|multicellular organismal development|IEA|GO:0007275//biological process|negative regulation of Wnt receptor signaling pathway|IEA|GO:0030178//cellular component|extracellular region|IEA|GO:0005576 |
| Unknown | Xl.11594.1.A1_at | None |
| RALDH2 | Xl.18999.1.A1_at | None |
| Prickle | Xl.7556.1.S1_at | cellular component|plasma membrane|IEA|GO:0005886//cellular component|membrane|IEA|GO:0016020//molecular function|protein binding|IPI|GO:0005515//molecular function|zinc ion binding|IEA|GO:0008270//molecular function|metal ion binding|IEA|GO:0046872 |
| ADMP | Xl.3809.1.A1_at | None |
| Unknown | Xl.1521.1.A1_at | None |
| Cytochrome B561 | Xl.11917.1.S1_at | biological process|electron transport|IEA|GO:0006118//biological process|transport|IEA|GO:0006810//cellular component|membrane|IEA|GO:0016020//cellular component|integral to membrane|IEA|GO:0016021//cellular component|cytoplasmic vesicle|IEA|GO:0031410//molecular function|iron ion binding|IEA|GO:0005506//molecular function|metal ion binding|IEA|GO:0046872 |
| Goosecoid | Xl.801.1.S1_at | cellular component|nucleus|IEA|GO:0005634//molecular function|DNA binding|IEA|GO:0003677//molecular function|transcription factor activity|IEA|GO:0003700//molecular function|sequence-specific DNA binding|IEA|GO:0043565 |
| FoxC1 | Xl.180.1.S1_at | biological process|regulation of transcription, DNA-dependent|IEA|GO:0006355//cellular component|nucleus|IEA|GO:0005634//molecular function|DNA binding|IEA|GO:0003677//molecular function|transcription factor activity|IEA|GO:0003700//molecular function|sequence-specific DNA binding|IEA|GO:0043565 |
| Noggin | Xl.834.1.S1_at | cellular component|extracellular region|IEA|GO:0005576 |
| Sprouty1 | Xl.10087.1.A1_Fat | None |
| Oct1 | Xl.1265.1.S1_at | biological process|transcription|IEA|GO:0006350//biological process|regulation of transcription, DNA-dependent|IEA|GO:0006355//biological process|regulation of transcription|IEA|GO:0045449//cellular component|nucleus|IEA|GO:0005634//molecular function|DNA binding|IEA|GO:0003677//molecular function|transcription factor activity|IEA|GO:0003700//molecular function|sequence-specific DNA binding|IEA|GO:0043565 |
| Rexp52 | Xl.3023.1.A1_at | None |
| Grb10 interacting protein2 | Xl.14208.1.A1_at | None |
| Putative methyltransferase | Xl.20056.1.S1_a_at | biological process|metabolic process|IEA|GO:0008152//molecular function|methyltransferase activity|IEA|GO:0008168 |
| Connexin 29 | Xl.8924.1.A1_at | biological process|cell communication|IEA|GO:0007154 |
| SMCT | Xl.6392.1.A1_at | biological process|transport|IEA|GO:0006810//biological process|ion transport|IEA|GO:0006811//biological process|sodium ion transport|IEA|GO:0006814//cellular component|membrane|IEA|GO:0016020//cellular component|integral to membrane|IEA|GO:0016021//molecular function|transporter activity|IEA|GO:0005215 |
| Weakly similar to Rab1 | Xl.3365.1.A1_at | None |
| Unknown | Xl.19961.1.S1_at | None |
| Moderately similar to Brain protein 44 | Xl.15887.1.S1_x_at | None |
| Ephrin receptor A2 | Xl.14496.1.A1_at | biological process|protein amino acid phosphorylation|IEA|GO:0006468//biological process|transmembrane receptor protein tyrosine kinase signaling pathway|IEA|GO:0007169//cellular component|membrane|IEA|GO:0016020//cellular component|integral to membrane|IEA|GO:0016021 |

IEA=Inferred from electronic annotation
