# Supplementary material for: Characterisation of the Fibroblast Growth Factor Dependent Transcriptome in Early Development
Source: PLoS One. 2009 Mar 31;4(3):e4951. doi: 10.1371/journal.pone.0004951 (PMC2659300; doi:10.1371/journal.pone.0004951)
Supplement: Table S13 — GO terms for genes negatively regulated by FGF signalling (0.04 MB DOC) [file pone.0004951.s015.doc]

**Table S13 GO terms for genes negatively regulated by FGF signalling**

| **Gene** | **Affymetrix**  **probe set** | **Go terms** |
| --- | --- | --- |
| XIRG protein | Xl.4965.1.S1_at | biological process|propionate catabolic process|IEA|GO:0019543//molecular function|2-methylcitrate dehydratase activity|IEA|GO:0047547 |
| PDGF A chain | Xl.841.3.S1_a_at | biological process|cell proliferation|IEA|GO:0008283//cellular component|extracellular region|IEA|GO:0005576//cellular component|membrane|IEA|GO:0016020//molecular function|growth factor activity|IEA|GO:0008083 |
| WIG-related | Xl.23988.1.S1_at | None |
| CP2-like transcription factor | Xl.16094.1.A1_at | None |
| Glucocorticoid inducible leucine zipper | Xl.12378.1.S1_at | biological process|regulation of transcription, DNA-dependent|IEA|GO:0006355//molecular function|transcription factor activity|IEA|GO:0003700 |
| Unknown | Xl.2077.1.A1_at | None |
| WIG | Xl.736.1.S1_at | cellular component|integral to membrane|IEA|GO:0016021 |
| XANF1 | Xl.131.1.S1_at | cellular component|nucleus|IEA|GO:0005634//molecular function|DNA binding|IEA|GO:0003677//molecular function|transcription factor activity|IEA|GO:0003700//molecular function|sequence-specific DNA binding|IEA|GO:0043565 |
| HES-related 1B | Xl.12126.1.S1_at | biological process|regulation of transcription, DNA-dependent|IEA|GO:0006355//biological process|regulation of transcription|IEA|GO:0045449//cellular component|nucleus|IEA|GO:0005634//molecular function|DNA binding|IEA|GO:0003677//molecular function|transcription regulator activity|IEA|GO:0030528 |
| Darmin | Xl.6024.1.S1_at | biological process|proteolysis|IEA|GO:0006508 |
| ODC2 | Xl.8949.1.S1_at | biological process|polyamine biosynthetic process|IEA|GO:0006596 |
| Unknown | Xl.11598.1.A1_at | None |
| Thioredoxin binding protein 2 | Xl.24749.1.A1_at | None |
| Unknown | Xl.25985.1.A1_at | None |
| Selenophosphate synthetase 1 | Xl.6522.1.A1_at | None |
| Adenosine deaminase | Xl.24155.1.A1_at | None |

IEA=Inferred from electronic annotation
