# Supplementary material for: Characterisation of the Fibroblast Growth Factor Dependent Transcriptome in Early Development
Source: PLoS One. 2009 Mar 31;4(3):e4951. doi: 10.1371/journal.pone.0004951 (PMC2659300; doi:10.1371/journal.pone.0004951)
Supplement: Table S14 — Expression of genes positively regulated by FGF signalling (0.38 MB DOC) [file pone.0004951.s016.doc]

**Table S14 Expression of genes positively regulated by FGF signalling**

| **Gene** | **Affymetrix probe set** | **Expression at early gastrula stage** |
| --- | --- | --- |
| Brachyury | Xl.514.1.S1_at | See Figure 1H |
| Egr1 | Xl.637.1.A1_at | Mesoderm with dorsal enrichment* |
| FoxD5A | Xl.642.1.S1_at | See Figure 4B |
| SIP1 | Xl.958.1.S2_at | Dorsal neuroectoderm* |
| Cdx4 | Xl.10269.1.S1_at | See Figure 1H |
| Esr5 | Xl.14524.1.S1_at | See Figure 2F |
| Purine phosphorylase | Xl.16206.1.A1_at | See Figure 2F |
| Marginal coil | Xl.5454.1.S1_at | Mesoderm with dorsal enrichment* |
| Paraxial protocadherin | Xl.6173.1.A1_at | Mesoderm with dorsal enrichment* |
| Glycogen phosphorylase | Xl.7815.1.A1_at | See Figure 2F |
| NADH dehydrogenase sub-unit | Xl.12993.1.A1_at | 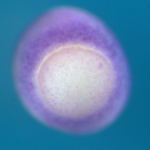 |
| FoxD3A | Xl.525.1.S1_at | Dorsal mesoderm* |
| G-coupled receptor P2Y5 | Xl.19933.1.S1_at | 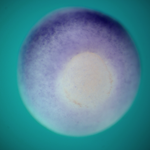 |
| Related to DC-STAMP domain receptor | Xl.15270.1.A1_at | 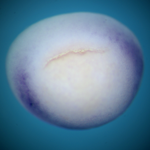 |
| Meso05 | Xl.7720.1.A1_at | 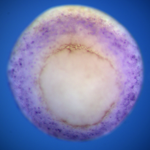 |
| **Gene** | **Affymetrix**  **probe set** | **Expression at early gastrula stage** |
| Uncharacterised protein C2orf32 | Xl.25136.1.A1_at | 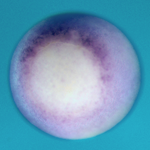 |
| Frzb1 | Xl.212.2.S1_a_at | See Figure 4B |
| XPO | Xl.5908.1.S1_s_at | Mesoderm* |
| Ephrin receptor A4 | Xl.13.2.A1_at | See Figure 2F |
| XSpr2 | Xl.2755.1.S1_a_at | 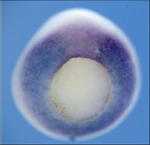 |
| Zic3a | Xl.7969.1.S1_at | Dorsal mesoderm and neuroectoderm* |
| Xiro3 | Xl.4522.1.S1_at | Dorsal neuroectoderm* |
| Gravin-like | Xl.3468.1.S1_at | 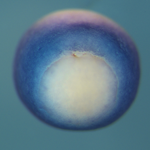 |
| Alkaline phosphatase | Xl.1299.1.S1_at | Unknown** |
| Apobec2 | Xl.5876.1.A1_a | See Figure 2F |
| p75-like fullback receptor | Xl.3540.1.S1_at | Mesoderm* |
| Wnt8 | Xl.49.1.S1_at | Ventro-lateral mesoderm* |
| Fructokinase-related protein | Xl.15623.1.A1_at | Unknown** |
| Crescent | Xl.619.1.S1_at | Dorsal mesoderm* |
| Pinhead | Xl.3529.1.A1_at | Mesoderm* |
| Wnt5b | Xl.11619.1.S1_at | Unknown** |
| Unknown | Xl.5479.1.A1_at | See Figure 2F |
| Retrotransposon protein 1a11 | Xl.3352.1.S1_at | Mesoderm* |
| FoxA4 | Xl.1082.1.S1_at | Dorsal mesoderm* |
| Mitotic phosphoprotein 67 | Xl.20772.1.A1_at | Unknown** |
| Cdx1 | Xl.23739.1.A1_at | Mesoderm with ventro-lateral enrichment* |
| Sprouty2 | Xl.11965.1.S1_at | Mesoderm* |
| DUSP5 | Xl.15374.1.A1_at | See Figure 2F |
| Chordin | Xl.3549.1.S1_at | Dorsal mesoderm* |
| MKP1 | Xl.2803.1.S1_at | See Figure 2F |
| Unknown | Xl.18179.1.S1_at | Unknown** |
| Xom | Xl.37.1.S1_at | Mesoderm with ventro-lateral enrichment* |
| **Gene** | **Affymetrix probe set** | **Expression at early gastrula stage** |
| Putative nucleolar GTP binding protein | Xl.14776.1.A1_at | 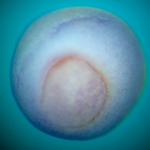 |
| Lin28a homologue | Xl.3418.1.A1_at | 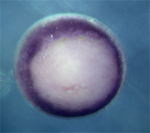 |
| Glut1 transporter | Xl.24121.1.A1_at | Mesoderm with dorsal enrichment* |
| Unknown | Xl.15382.1.A1_at | Unknown** |
| Dkk1 | Xl.251.1.S1_at | Dorsal mesoderm* |
| Unknown | Xl.11594.1.A1_at | Unknown** |
| RALDH2 | Xl.18999.1.A1_at | Mesoderm with dorsal enrichment* |
| Prickle | Xl.7556.1.S1_at | Mesoderm with dorsal enrichment* |
| ADMP | Xl.3809.1.A1_at | Dorsal mesoderm* |
| Unknown | Xl.1521.1.A1_at | Unknown** |
| Cytochrome B561 | Xl.11917.1.S1_at | Unknown** |
| Goosecoid | Xl.801.1.S1_at | Dorsal mesoderm* |
| FoxC1 | Xl.180.1.S1_at | Mesoderm with dorsal exclusion* |
| Noggin | Xl.834.1.S1_at | Dorsal mesoderm* |
| Sprouty1 | Xl.10087.1.A1_Fat | Mesoderm* |
| Oct1 | Xl.1265.1.S1_at | Ectoderm and mesoderm* |
| Rexp52 | Xl.3023.1.A1_at | General |
| Grb10 interacting protein2 | Xl.14208.1.A1_at | Unknown** |
| Putative methyltransferase | Xl.20056.1.S1_a_at | See Figure 2F |
| Connexin 29 | Xl.8924.1.A1_at | Unknown** |
| SMCT | Xl.6392.1.A1_at | Unknown** |
| Weakly similar to Rab1 | Xl.3365.1.A1_at | Unknown** |
| Unknown | Xl.19961.1.S1_at | Unknown** |
| Moderately similar to Brain protein 44 | Xl.15887.1.S1_x_at | Unknown** |
| Ephrin receptor A2 | Xl.14496.1.A1_at | Unknown** |

*****=Based upon published expression data.

**=Expression pattern not investigated.
