# Supplementary material for: Characterisation of the Fibroblast Growth Factor Dependent Transcriptome in Early Development
Source: PLoS One. 2009 Mar 31;4(3):e4951. doi: 10.1371/journal.pone.0004951 (PMC2659300; doi:10.1371/journal.pone.0004951)
Supplement: Table S15 — In situ clone data (0.06 MB DOC) [file pone.0004951.s017.doc]

**Table S15 In situ clone data**

| **cDNA** | **GenBank**  **Accession** | **Source** | **Notes** |
| --- | --- | --- | --- |
| Apobec2 | AW766385 | Geneservice | Image clone 3200688 |
| Brachyury | M77243 | Jim Smith | [1] |
| Cdx4 | U02034 | David Kimelman | [2] |
| C2orf32 | BJ15151 | NIBB^2^ | Clone xl170m14 |
| Chordin | BC077767 | Geneservice^1^ | Image clone 5161617 |
| CP2-like | BJ068545 | NIBB^2^ | Clone xl107a09 |
| DC-stamp related | BJ037349 | NIBB^2^ | Clone xl040d20 |
| DUSP5 | BC074485 | Geneservice^1^ | Image clone 6952431 |
| Ephrin receptor A4 | BC043626 | Geneservice^1^ | Image clone 5537382 |
| Esr5 | BJ624157 | NIBB^2^ | Clone xl207h08 |
| FoxD5 | BJ627201 | NIBB^2^ | Clone xl217f11 |
| Frzb1 | U78598 | Malcolm Moos | [3] |
| Glucocorticoid  Inducible leucine zipper | BC043841 | Geneservice^1^ | IMAGE clone 5570146 |
| Glycogen phosphorylase | BJ056085 | NIBB^2^ | Clone xl008i01 |
| Gravin-like | BJ622102 | NIBB^2^ | Clone xl199m22 |
| Meso5 | DC113614 | NIBB^2^ | Clone xl266d07 |
| Methyltransferase | BJ100128 | NIBB^2^ | Clone xl173h17 |
| MKP1 | BJ072620 | NIBB^2^ | Clone xl099d04 |
| MyoD | X16106 | John Gurdon | [4] |
| Lin28a | BJ043066 | NIBB^2^ | Clone xl033i12 |
| NADH dehydrogenase | BJ066761 | NIBB^2^ | Clone xl088c03 |
| Nucleolar GTP binding protein | DC124229 | NIBB^2^ | Clone xl304p12 |
| P2Y5 | BQ400802 | Geneservice^1^ | IMAGE clone 4969022 |
| Purine phosphorylase | BJ029844 | NIBB^2^ | Clone xl013l05 |
| Wig-related | BJ044287 | NIBB^2^ | Clone xl012n09 |
| Xl.5479 | BJ092401 | NIBB^2^ | Clone xl087c12 |
| XSpr2 | AY062263 | NIBB2 | Clone xl049o23 |

**^1^Geneservice Ltd: http://www.geneservice.co.uk/home/**

**^2^NIBB: http://xenopus.nibb.ac.jp/**

**References**

1. Smith JC, Price BMJ, Green JBA, Weigel D, Herrmann BG (1991) Expression of a Xenopus homolog of Brachyury (T) is an immediate-early response to mesoderm induction. cell 67: 79-87.

2. Northrop JL, Kimelman D (1994) Dorsal-ventral differences in Xcad-3 expression in response to FGF-mediated induction in Xenopus. developmental biology 161: 490-503.

3. Wang S, Krinks M, Lin K, Luyten FP, Moos M, Jr. (1997) Frzb, a secreted protein expressed in the Spemann organizer, binds and inhibits Wnt-8. cell 88: 757-766.

4. Hopwood ND, Pluck A, Gurdon JB (1989) MyoD expression in the forming somites is an early response to mesoderm induction in Xenopus embryos. embo journal 8: 3409-3417.
