# Supplementary material for: Characterisation of the Fibroblast Growth Factor Dependent Transcriptome in Early Development
Source: PLoS One. 2009 Mar 31;4(3):e4951. doi: 10.1371/journal.pone.0004951 (PMC2659300; doi:10.1371/journal.pone.0004951)
Supplement: Table S16 — RNAase protection probe data (0.04 MB DOC) [file pone.0004951.s018.doc]

**Table S16 RNAase protection probe data**

| **cDNA** | **Probe size** | **Transcribe** | **Linearize** | **Notes** |
| --- | --- | --- | --- | --- |
| Cdx4 | 400bp | T7 | EcoRI | (Pownall et al., 1996) |
| Esr5 | 291bp | SP6 | NcoI | PCR subclone of BJ624157 |
| Purine phosphorylase | 224bp | T7 | SalI | PCR subclone of BJ029844 |
| Glycogen phosphorylase | 237bp | T7 | SalI | PCR subclone of BJ056085 |
| Ephrin receptor A4 | 220bp | T7 | SalI | PCR subclone of BC043626 |
| Xl.5479 | 403bp | T7 | SalI | PCR subclone of BJ092401 |
| DUSP5 | 348bp | T7 | SalI | PCR subclone of BC068545 |
| MKP1 | 400bp | T7 | XhoI | PCR subclone of BJ072620 |
| Methyltransferase | 339bp | T7 | SalI | PCR subclone of BJ100128 |
| ODC | 91bp | T7 | BglII | [1] |
| MyoD | 360bp | SP6 | EcoRI | [2] |
| Brachyury | 220bp | T7 | SspI | [3] |

**References**

1. Isaacs HV, Tannahill D, Slack JMW (1992) Expression of a novel FGF in the Xenopus embryo. A new candidate inducing factor for mesoderm formation and anteroposterior specification. development 114: 711-720.

2. Harvey RP (1991) Widespread expression of MyoD genes in Xenopus embryos is amplified in presumptive muscle as a delayed response to mesoderm induction. proceedings of the national academy of sciences of the united states of america 88: 9198-9202.

3. Isaacs HV, Pownall ME, Slack JMW (1994) eFGF regulates Xbra expression during Xenopus gastrulation. embo journal 13: 4469-4481.
